# An integrated reservoir operation framework for enhanced water resources planning

**DOI:** 10.1038/s41598-023-49107-z

**Published:** 2023-12-08

**Authors:** Sonam Sandeep Dash, Bhabagrahi Sahoo, Narendra Singh Raghuwanshi

**Affiliations:** 1 School of Civil Engineering, University College Dublin, Dublin, Ireland; 2 School of Water Resources, Indian Institute of Technology Kharagpur, Kharagpur, India; 3 Agricultural and Food Engineering Department, Indian Institute of Technology Kharagpur, Kharagpur, India

**Keywords:** Hydrology, Climate-change adaptation, Climate-change impacts

## Abstract

Climate change induced spatiotemporal variation in global water availability modifies the proposed design criteria of water infrastructure structures like dams and reservoirs. Although reservoir operation is treated as a potential adaptation option, obsolescence of existing operation rules in the climate change scenarios could cause devastating situation through faulty water management practices. Presently onboard simulation–optimization based reservoir operation schemes fail to capture the uncertainty involved in the climate change scenario. Hence, there is a need to identify the limiting application scenario of the existing reservoir operation rule, and subsequently, revise the operation framework to address the future supply–demand uncertainty adequately. This research develops an integrated Soil and Water Assessment Tool (SWAT) (hydrologic), HEC-ResSim (hydraulic), and genetic algorithm (GA) (optimization) based adaptive reservoir operation framework, which is competent enough in accounting the future supply–demand uncertainty. Incorporation of the newly proposed environmental flow assessment approach in the reservoir operation would assist the decision makers in guiding the reservoir release for maintaining the water quality and sustenance of the downstream aquatic species. Certainly, corresponding to the existing operation rules under both the baseline and future climate change scenarios of RCP 4.5 and 8.5, the developed SWAT-HEC-ResSim-GA based reservoir operation scheme could improve the performance of the Kangsabati reservoir with the time and volume reliability estimates of 0.631 and 0.736, respectively. Conclusively, the developed approach in this study could be the best feasible alternative for hydrologic characterization in complex reservoir catchment-command regions with the option for enhanced reservoir planning in global catchment-command regions.

## Introduction

The dearth of water in conjunction with climate change imparts severe threat to the user and decision-making community^1^. The global water managers are becoming more concerned about the potential impacts of climate change on the water supply–demand dynamics^2,3^. The ever-increasing temperature scenario because of greenhouse effect results in increased evaporation loss from the reservoir than normal condition. Global warming and precipitation fluctuations could result in adverse flood or drought conditions, alteration in environmental flow (E-flow), and increased water demand by the stakeholders. Since agriculture accounts for 70% of global water withdrawals, the climate change derived future crop-water alteration could adversely affect the supply–demand uncertainty in the arid and semi-arid regions of globe^4^. Under these plausible future climate change scenarios, increasing water supply by dam construction and improved reservoir capacity may be the feasible solutions for sustainable management of water demand leading to global socioeconomic development^5^. Hence, evaluation of the existing reservoir operation policies and its judicious modification could provide the policy makers with a more dynamic insight to alleviate the adverse effects of climate change on water availability.

As per the IPCC working group II report, many countries across the globe have started proposing adaptive water resources management plan based on projected climate change and the involved uncertainty. Eventually, employment of such adaptive rules takes care of the underlying risk, system customization ability, and subsequently, modifies the decision rule throughout the analysis time horizon. The major advantage of a risk-based framework is that it can quantify the actual impact of climate change and simultaneously modify the decision-making process. In impact assessment studies, for the estimation of projected future climate scenario, General circulation Model (GCM) seems to be the best possible alternative^6^. Recently, the CMIP5 GCM projections are available at a coarser resolution, ranging from 0.5° to 4° with four RCP scenarios of 2.6, 4.5, 6.0, and 8.5^7^. Considering the degree of coarseness existing with the raw GCM outputs, direct application in impact assessment studies may raise questions over the accuracy of the analysis^8–10^. To deal with the inherent uncertainty involved with individual GCMs, a multi-model ensemble technique that combines multiple GCM outputs to yield a single output has been well adopted in the past hydro-climatological studies^11^.

The contrasting supply–demand scenario during the monsoon season and lean period poses challenges in terms of maintaining the structural stability of reservoir and altered flow regime, making the reservoir rule formulation more intricate. The conventional optimization-based reservoir operation techniques like linear/nonlinear/dynamic programming and heuristic approaches perform poorly under reservoir operation having conflicting objective functions, resulting erroneous policy formulation^12,13^. The dynamic programing-based reservoir operation approaches are usually solved by integrating solutions of smaller sub-problems; hence, only suitable for short-range applications like hourly- or daily-scale^14^. Adoption of hydrological quantification framework and reservoir operation framework individually may not yield the actual feedback in the complex river-reservoir system, which is ignored in previous reservoir simulation studies^15,16^. The current reservoir operation schemes do not consider the environmental flow (E-flow) component for demand computation, resulting in an underestimation of the total demand^17^. The future climate change impact studies reported that increased annual temperature, decreased streamflow resulted increased agricultural water demand than the other form of demand in the climate change scenario^1^. Further, the outcomes of modelling revealed lower reliability and increased vulnerability of the reservoir operation under climate change scenarios. The Kangsabati reservoir located in the semi-arid region of eastern part of India could be subjected to significant variability in the reservoir inflow and different demand components. The accurate quantification of these components under varying climate change scenario could behave as an early warning system for the competent reservoir management authorities to take necessary adaptation measures. Nevertheless, assessment of future water availability in complex integrated river-reservoir catchment-command system using the conventional approaches is quite cumbersome making the reservoir performance assessment more intricate. Given a series of odds, the water managers may realize the importance to move beyond the conventional scenario-specific impact analysis approach to an adaptive, risk-based planning approach that can render the effects from a spectrum of plausible future climate conditions. This necessitates adoption of a reliable integrated modelling framework, which can simultaneously account the catchment-hydrology and reservoir hydraulic characteristics in formulating an adaptive reservoir operation rule.

In light of this, the research gaps concerning to reservoir operation under future climate change scenario are identified as: (1) operational reservoir policy lacks provision for futuristic corrections; (2) accurate quantification of future water distribution dynamics in multifaceted river-reservoir catchment command location is yet to be analyzed; (3) incorporation of environmental flow component in the reservoir operation rule formulation is ignored in all past studies; and (4) conventional optimization only approach may not yield satisfactory reservoir operation in complex catchment-command regions. In this context, the present study tries to address the following research questions: (i) What will be the water availability scenario in the Kangsabati reservoir under the future climate change scenario?; and (ii) Will the existing operation rule of the Kangsabati reservoir be competent enough in satisfying the supply–demand dynamics under climate change scenarios?; (iii) How effectively the inclusion of E-flow component will describe the supply–demand dynamics over the study location?; and (iv) Will the proposed operation rule assist the decision-makers in planning a sustainable water management policy in future climate change scenario? To perform this assignment, the specific objective of this study includes: (1) to develop an integrated SWAT-HEC-ResSim-GA based reservoir operation framework for optimized fulfilment of various demand components under the baseline scenario; (2) to quantify the future reservoir inflow and different demand components over the study area; and (3) to evaluate the applicability of the proposed reservoir operation rule under future climate change scenario; and (4) to quantify the associated uncertainty in the GCM projections, downscaling approach, and the adopted modelling framework.

The enhanced SWAT-based hydrological framework adopted herein is an advance over the conventional SWAT, with improved conceptualization of irrigation application and streamflow estimation under dynamic vadose zone process of agricultural catchment^18^. The physically based HEC-ResSim based reservoir simulation model has not been coupled with the enhanced SWAT model in any past studies; thereby signifying a robust methodology to study the integrated effect of river and reservoir on catchment water distribution. Moreover, the proposed dynamic hydrological and reservoir simulation framework enables the user to account for the future climate and changed reservoir hydraulic characteristic for updated rule curve formulation, which is absent in the formerly proposed reservoir operation framework. In essence, the established SWAT-HEC-ResSim-GA framework serves as the foundation for a resilient reservoir management system with better understanding of complex system, offering the ability to assess uncertainties originating from various sources at different phases of decision-making, representing a novel approach on its own. The proposed physically based modelling approach can effectively assess the future water availability in complex river-reservoir system with least uncertainty. Overall, the performed research can behave as a suit of policy making tool for the decision makers, enabling them to ubiquitously detect the involved uncertainty across various decision-making phases and can be well applied across any global river-reservoir catchment.

## Material and methods

### Hydrologic and reservoir simulation model framework

**Figure 1:**
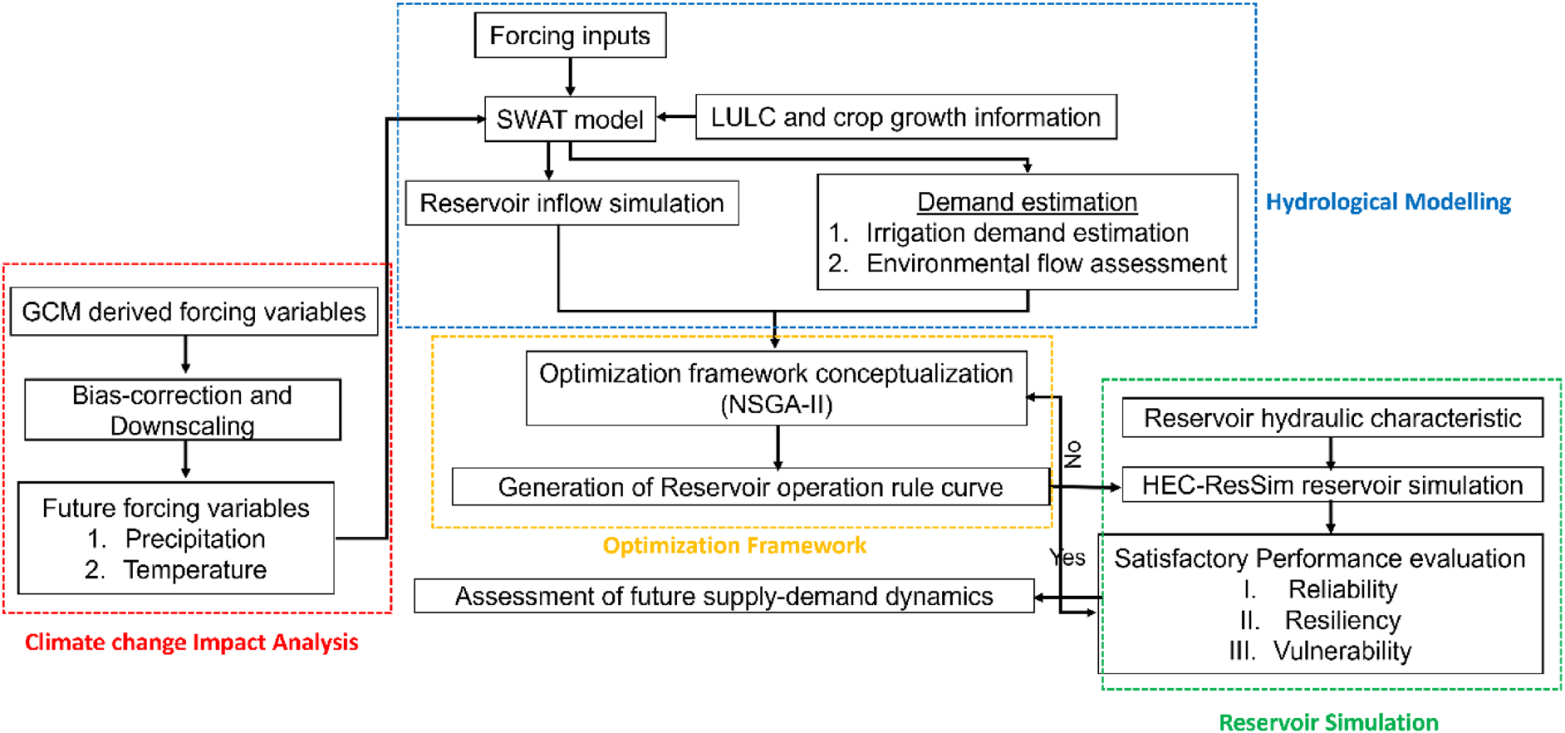
Integrated reservoir operation framework under future climate change scenario.

The integrated reservoir operation rule showing the detailed procedure of integration of different components in the proposed framework is presented in Fig. 1.

#### SWAT hydrological model

The Soil and Water Assessment Tool (SWAT) is a catchment-scale, semi-distributed, and daily/sub-daily-scale model, extensively employed for hydrologic quantification of river catchments^19^. As a process-based model, the SWAT utilizes different conceptualizations to understand the hydrological flux generation and dynamics inside the catchment. The SWAT conceptualization is exclusively centered on the principle of mass conservation, momentum conservation, and energy conservation^20^. The SWAT employs the convention curve number (CN) and pothole-based streamflow simulation approach for the non-paddy and paddy growing regions, respectively. However, considering the inherent dynamics in the paddy field hydrological behavior, it has undergone substantial modifications^21,22^. To account for the complex paddy growing regions located in the integrated river-reservoir catchment regions, the most recent physically based enhanced SWAT-Pothole approach proposed by^18^ has been incorporated in the present study. Further, to quantify the crop water requirement of the concerned study location, the embedded FAO-56 Penman–Monteith (PM) in the SWAT was chosen for both baseline and future climate change scenarios. The detailed workflow of the SWAT model for reservoir inflow simulation is presented in Fig. A1.

#### Reservoir simulation framework

The HEC-ResSim, which is an advancement over the previously proposed conceptual HEC-5 model, is adopted in the present study for long-term continuous reservoir inflow, outflow, and storage simulation^23^. The HEC-ResSim assists in irrigation planning and flood control operations in complex catchment-command regions. In practice, the HEC-ResSim acts as a combination of both hydrologic and hydraulic conceptualization, and subsequently, assists in integrated supply–demand management^23^. The HEC-ResSim consists of multiple sub-routines including, watershed module, reservoir network module, and reservoir simulation module, where each module serves unique purpose.

The reservoir system is represented by four types of elements, viz., junctions, routing reaches, reservoir, and diversions in the HEC-ResSim model conceptualization. The actual operational rule curves are defined for the decision-making process in governing the release schedule of the reservoir. In real world conditions, the flow requirement and the related constraints are the explicit function of the current state of the reservoir pool. Hence, the HEC-ResSim discretizes the pool into varying elevation bands; thereby assigning different sets of prioritized rule curve to individual operating zones for guiding the release accordingly. Moreover, reservoir simulation modelling in the HEC-ResSim domain requires to define the target elevation level exclusively. The guide curve, i.e., the dividing line between the upper zone (Flood-control pool) and lower zone (conservation pool) of reservoir, which is a function of time primarily decides the release decision logic of the reservoir. When the pool elevation reaches above the guide curve, the additional water is released through the emergency spillway in the form of uncontrolled release and upon attaining the lower level of guide curve, considering the user-defined rule curve less water is set to release out of the pool. Further, a series of connecting reservoirs can be modelled through complex conceptualization of the river-reservoir system through defining nested reservoir operation rule curves across all the reservoirs located in the model domain. The detailed workflow of the reservoir outflow simulation by the HEC-ResSim reservoir simulation is presented in Fig. A2.

### Reservoir operation rule formulation

**Figure 2:**
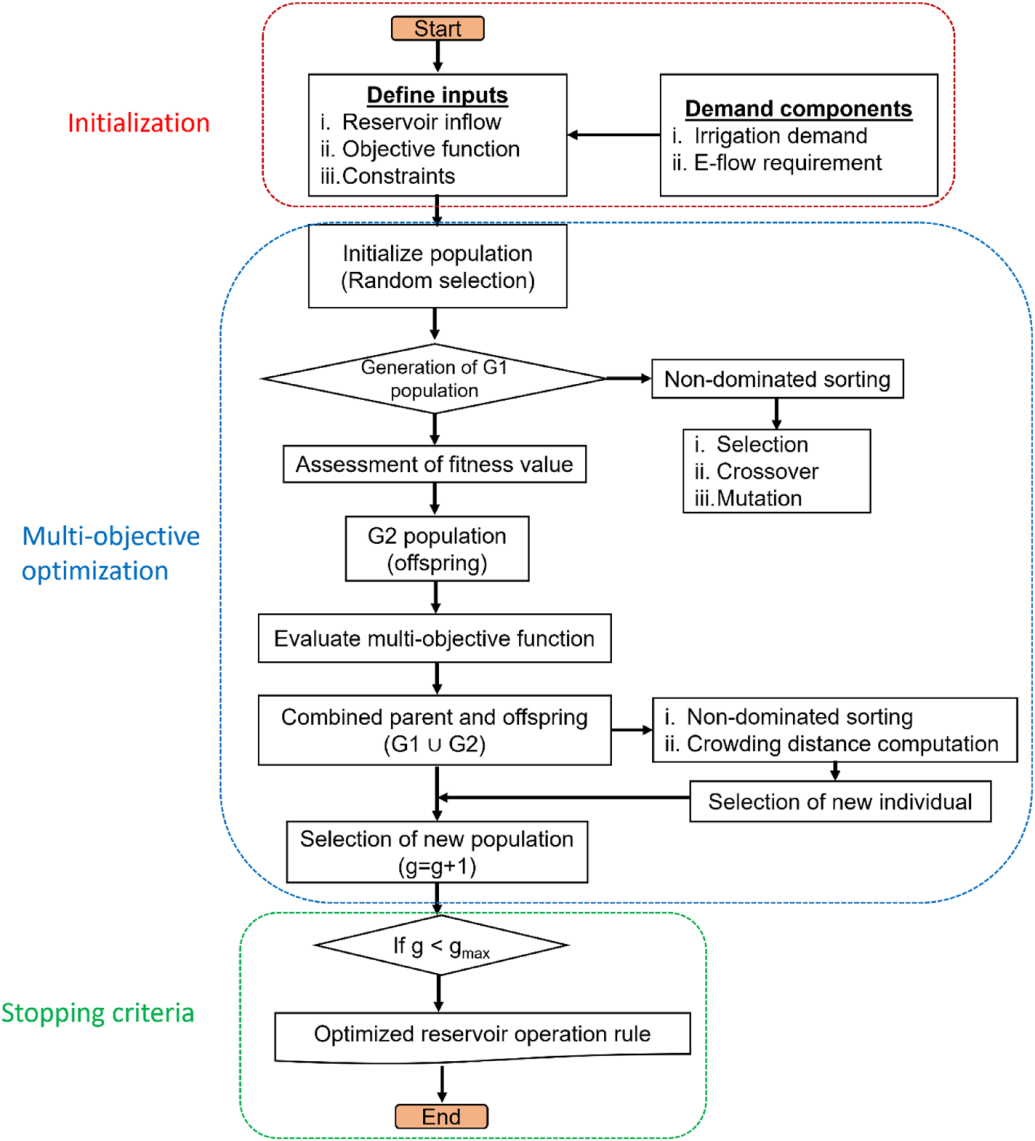
Adopted reservoir optimization framework in the present study.

The proposed integrated simulation–optimization based framework for the Kangsabati reservoir is characterized by the given objective functions, viz., minimization of reservoir release and maximizing the capacity of reservoir storage to assist in flood governance and fulfilment of water demand, respectively. The present water level of the reservoir is chosen as the decision variable in the conceptualization. The detailed process flowchart for the performed optimization study is presented in Fig. 2. The expression of the proposed objective functions is given below as follows:

The minimization in the water supply deficit (f_1_) is given by:

1 $${f}_{1}=Minimize\sum_{t=1}^{12}\left[{\left({R}_{t}-{D}_{t}\right)}^{2}-{\left({S}_{t}-{S}_{T}\right)}^{2}\right]$$

where ${R}_{t}$ is the water release to meet demand (MCM); ${D}_{t}$ is the total water demand at downstream (MCM); ${S}_{t}$ is the volume of water storage at the beginning of reservoir simulation in the period ‘t’ (MCM); and ${S}_{T}$ is the target reservoir storage in period ‘t’ (MCM).

The second objective function maximizing the reservoir storage (f_2_) is described as:

2 $${f}_{2}=Minimize\sum_{t=1}^{12}Max\left\{{{Q}_{t}}^{D}-{{Q}_{t}}^{Th},0\right\}$$

where ${{Q}_{t}}^{D}$ is the demand specific reservoir in month ‘t’ (MCM); ${{Q}_{t}}^{Th}$ is the downstream discharge threshold resulting flood control (MCM).

The following constraints are defined to address the proposed objective functions.

The water balance constraint is given by:

3 $${S}_{t+1}={S}_{t}+{I}_{t}-{R}_{t}-{E}_{t}-{O}_{t}-{P}_{t}, t=\mathrm{1,2},\dots .,12$$

where ${S}_{t+1}$ is the water storage in the end of tth month (MCM); ${I}_{t}$ is the inflow to reservoir in the tth month (MCM); ${E}_{t}$ is the reservoir evaporation loss in the tth month (MCM); ${O}_{t}$ is reservoir outflow in the tth month (MCM); and ${P}_{t}$ is the reservoir seepage loss in the tth month (MCM).

Further, the active storage and designed storage in the reservoir have been in tth month introduced by means of the storage constraint as given below:

4 $${S}_{dead}\le {S}_{t}\le {S}_{max}, t=\mathrm{1,2},\dots .,12$$

where ${S}_{dead}$ and ${S}_{max}$ are the reservoir dead storage capacity and maximum possible reservoir storage capacity, respectively.

The amount of water that could be released in each month corresponding to the various demand component is depicted as the release constraint, expressed as:

5 $${R}_{t}\le {D}_{t}, t=\mathrm{1,2},\dots .12$$

The excess water flow from the reservoir under release exceedance condition is termed as the overflow constraint, given as:

6 $${O}_{t}={S}_{t+1}-{S}_{max}$$

### Climate change impact assessment

The climate change impact assessment was carried out in this study by forcing the GCM derived precipitation and temperature estimates into the previously calibrated and validated SWAT model. Since the RCP 2.6 corresponds to the least GHG emission scenario, the corresponding rainfall and temperature scenarios may not be the true representative of the plausible climate condition of the KRB. Moreover, the RCP 6.0 as a moderate GHG emission scenario poses no significance for the analysis. Hence, in this study, the CMIP5 derived five GCMs were chosen for projecting the future climatic conditions of the KRB under two RCP scenarios of 4.5 and 8.5, depicting the lowest and highest plausible radiative forcing conditions, respectively.

The inherent uncertainty in the climate model leads to the adoption of substantial correction in the forcing variables prior to the model specific operation. Moreover, the coarser resolution of raw GCM outputs depicts their inability to represent the actual erratic variation of the forcing variables like precipitation and temperature, which is obvious in larger catchment-scale conditions. Hence, the five GCMs were appropriately bias corrected and downscaled with reference to suitable observed meteorological variables using the statistical downscaling approach^24^ as discussed below.

#### Selection of general circulation model (GCM)

In general, the climate change impact assessment is carried out by forcing the GCM derived precipitation and temperature estimates to the previously calibrated and validated hydrological or reservoir simulation model. In this study, the CMIP5 derived nine GCMs were chosen originally for projecting the future climatic conditions of the KRB under two RCP scenarios of 4.5 and 8.5. The source of origin and spatial resolution of the nine chosen GCMs are presented in Table A1 (Appendix section). Subsequently, the performance evaluation of the GCM with respect to the observed rainfall and temperature estimates at two representative locations of reservoir upstream and command area, five GCMs are shortlisted for the construction of multi-model ensemble and corresponding analysis.

As the physical process dynamics of different GCMs vary significantly, it is quite difficult to draw a solid conclusion about the regional climatology based on the individual GCM output. In this context, a suitable climate ensemble approach was adopted to construct an ensemble of multiple GCMs, hereafter, referred as multi-model ensembles (MME), which would behave as a representative future climate scenario for the KRB. However, all the GCMs may not represent the true climatology of a region; hence, selection of appropriate GCMs for the construction of MME is an important step. To identify the suitable GCMs for constructing the MME, the statistical relationship between the gridded IMD rainfall/temperature and individual GCM outputs including mean, standard deviation, root mean square deviation (RMSD), and PBIAS were computed which are illustrated in the form of Taylor diagrams (Figs. A3–A5). As envisaged from Fig. A3, the regional rainfall scenario is best reproduced by the MIROC5 GCM and the remaining RCMs exhibit almost similar estimates of standard deviation and correlation coefficient. However, the magnitude of daily rainfall is the best indicative of GCM performance, and the deviation of the GCM rainfall estimates from the observed rainfall was quantified in terms of the PBIAS. The higher PBIAS (> 10%) of the four GCMs, i.e., GFDL-CM3, IPSL-CM5A-LR, IPSL-CM5A-MR, and HadGEM2-ES in reproducing the daily rainfall led to non-consideration of these four GCMs in creating the MME of future rainfall. Similarly, the PBIAS estimates for reproducing the observed daily T_min_ and T_max_ variables in the KRB showed relatively higher value for the above said four GCMs; hence, excluded from the subsequent analysis.

#### Downscaling and bias-correction of GCM

The grid-scale downscaling performed in this study consisted of 99 spatial grid locations encompassing the whole Kangsabati River Basin (KRB). The GCMs chosen in this study are of varying spatial resolution, whereas the NCEP predictors are available at a fixed spatial resolution of 2.5° $\times$ 2.5°. Hence, using the bilinear interpolation technique, the GCMs were re-gridded to the NCEP grids to avoid the potential mismatch in the predictor grids.

Predictors are the GCM simulated climatic variables present in a region, which are extensively used in the statistical downscaling of the desired local-scale meteorological variables, such as precipitation and temperature, referred as the predictand. Since the predictor-predictand interrelationship varies across different seasons following spatiotemporal variation of atmospheric circulation, a seasonal stratification approach was adopted to perform the analysis individually in three different seasons of pre-monsoon (March–May), monsoon (June–September), and post-monsoon (October-February). Since the GCM based predictor variables are associated with systematic biases in their mean and variance magnitudes relative to the NCEP predictors, to reduce the extent of bias in the analysis, a standardization approach was followed as given by:

7 $${P}_{standardized}=\frac{{P}_{actual}-{P}_{mean}}{{P}_{standard deviation}}$$

where ${P}_{standardized}$ is the standardized predictor variable, ${P}_{actual}$ is the actual predictor variable, and ${P}_{mean}$ and ${P}_{standard deviation}$ are the mean and standard deviation of predictor estimates, respectively.

The readily available IMD gridded dataset at a spatial resolution of 0.25° $\times$ 0.25° was used as the observed dataset for carrying out the bias-correction for more realistic future projections in the KRB. The widely adopted Quantile Mapping (QM) bias-correction technique, as suggested by^25,26^ was used herein for the bias correction of daily rainfall, minimum temperature (T_min_), and maximum temperature (T_max_). To perform the bias-correction, the complete historical time period of 55 years (1951–2005) was divided into 1951–1975 (25 years) and 1976–2005 (30 years) as the testing and training periods, respectively.

In this study, a parametric multiple linear regression (MLR) and non-parametric Kernel regression (KR) approaches were used to establish the functional relationship between the predictor and predictand. In the statistical learning approach, an input was matched to the output at every point. In the MLR approach, a linear relation is fitted between the desired predictand (rainfall/temperature) and NCEP/GCM derived predictor variables. Conversely, the non-linear kernel regression approach estimates the conditional expectation of random variable by means of a weighted average technique; wherein the present study adopts a Gaussian kernel function having mean and standard deviation values of 0 and 1, respectively. To calibrate and validate the downscaling model, 30 (1976–2005) and 25 (1951–1975) years of training and testing periods were chosen, respectively. The calibration of the Kernel regression model was characterized by the selection of suitable smoothing parameter which forms the primary step of the kernel estimation. Further, the tuned calibration parameters performed satisfactorily during the validation period were treated as the transfer function and applied on the GCM predictors to estimate the future climate projections for the respective grid locations.

### Assessment of future water availability scenario

Owing to the significant variation in the KRB future climatology, the water balance components are subjected to substantial alteration. The future water availability in the KRB is studied at two representative locations of rain-fed upstream and downstream command region during the future time horizons of Near-future (2020–2045) (NF), Mid-future (2046–2071) (MF), and Far-future (2072–2099) (FF). To assess the future water availability in the KRB, the following stepwise procedure was followed:

i. The pre-calibrated and validated SWAT model was forced with the bias-corrected weather inputs from the five GCMs and multi model ensemble (MME) for the two representative scenarios of RCP 4.5 and 8.5.
ii. The hydrological simulations were performed over the three pre-defined future time horizons, and subsequently, the monthly-scale estimates of the two major water balance components, viz., streamflow and ET were estimated. Because of this alteration, the corresponding changes in the two associated water balance components, viz., soil moisture storage and groundwater recharge were assessed for the above said future timescales.
iii. The changes in the future water balance components corresponding to the baseline projections were analyzed.
iv. Further, the trend of the streamflow and ET components were estimated using the Mann–Kendall (MK) and Sen’s slope test statistics as given below.

The Mann–Kendall (MK) test was formulated by^27^ and its mathematical expression is given by:

8 $$S=\sum_{i=1}^{n-1}\sum_{j=i+1}^{n}sgn \left({x}_{j}-{x}_{i}\right)$$

where *n* is the length of data series, ${x}_{i}$ and ${x}_{j}$ are the data values in the time series *i* and *j* (*j* > *i*), respectively, and $sgn \left({x}_{j}-{x}_{i}\right)$ is the sign function expressed as^27^:

9 $$sgn\left({x}_{j}-{x}_{i}\right)=\left\{\begin{array}{c}+1,\quad if \,{x}_{j}-{x}_{i}>0\\ 0,\quad if\, {x}_{j}-{x}_{i}=0 \\ -1,\quad if\, {x}_{j}-{x}_{i}<0\end{array}\right.$$

The variance is computed as:

10 $$Var\left(S\right)=\frac{n\left(n-1\right)\left(2n+5\right)-({\sum }_{i=1}^{P}{t}_{i}\left({t}_{i}-1\right)\left({2t}_{i}+5\right))}{18}$$

where $P$ is the number of tied pairs and ${t}_{i}$ is the number of data values in the $P$ ^th^ group. If there are no tied groups, this summary process may be ignored. A tied group is a set of sample data having the same value.

The significance test is carried out using the Z-score given by:

11 $$Z_{calculated} = \left\{ \begin{gathered} \frac{S - 1}{{\sqrt {Var(S)} }} \, \quad if \, S > 0 \hfill \\ { 0 }\quad if \, S = 0 \hfill \\ \frac{S + 1}{{\sqrt {Var(S)} }} \, \quad if \, S < 0 \, \hfill \\ \end{gathered} \right. \,$$

If the $Z_{calculated} > Z_{tabulated}$, null hypothesis gets rejected, and treated as statistically significant trend. Alternatively, the upward or downward trends in a time series is known by the positive or negative value of the $Z_{calculated}$. For this study, the significance level, $\alpha$ = 5% (Z_0.05_ =  ± 1.96), which is the threshold cut-off for rejecting the null hypothesis.

### Assessment of future water demand scenarios in the basin

As the Kangsabati reservoir is not meant to fulfill the domestic water needs under the baseline scenario, there is no scope left for the reservoir to address this issue under the future climate change scenarios. Hence, like the baseline scenario, the future water demand is assumed to be from two major water consumption sectors, i.e., irrigation water requirement and environmental flow requirement. The methodology to estimate the various water demand components are given below.

#### Irrigation water demand assessment under future climate change scenarios

The irrigation water demands in the Kangsabati command area under future climate change scenarios was assessed assuming that, as compared to the baseline period, there would not be any significant change in the crop calendar and crop growing locations in future. For computing the reference evapotranspiration (*ET*_0_) during the baseline scenario, the FAO-56 Penman–Monteith (PM) method was used for which temperature, wind speed, and solar radiation are the input variables. However, in the future climate change scenario, the GCM derived outcomes only provide the information of rainfall, and minimum and maximum temperatures. Contemplating the accuracy of the FAO-56 PM approach, an indirect mean was adopted to derive the essential missing meteorological variables for *ET*_0_ estimation. Under limited data availability conditions, to estimate missing information on relative humidity (RH), the actual vapor pressure can be computed with the assumption that the dew point temperature is very close to the mean annual temperature as:

12 $${e}_{a}=0.611exp\frac{17.27{T}_{min}}{{T}_{min}+237.3}$$

Subsequently, RH is estimated as:

13 $$RH=\frac{{e}_{a}}{{e}_{s}}\times 100$$

where ${e}_{s}$ is the saturated vapour pressure (kPa).

The estimates of daily solar radiation can be derived using the minimum and maximum temperatures under different RCP scenarios as^28^:

14 $${R}_{s}=0.16{R}_{a}\sqrt{{T}_{max}-{T}_{min}}$$

where ${R}_{s}$ is the actual solar radiation (MJ m^−2^ day^−1^) and its value is a function of the latitude and day of the year which becomes a location specific constant value, and ${R}_{a}$ is the extraterrestrial radiation estimated as:

15 $${R}_{a}=\frac{1440}{\pi }{S}_{c}\times {I}_{d}\left({S}_{ha}\times {\text{sin}}{S}_{l}\times {\text{sin}}{S}_{d}+{\text{cos}}{S}_{l}\times {\text{cos}}{S}_{d}\times {\text{sin}}{S}_{ha}\right)$$

where ${S}_{c}$ is the solar constant (0.082 MJ m^−2^ day^−1^); ${I}_{d}$ is the inverse relative distance estimated as:

16 $${I}_{d}=1+0.033\times {\text{cos}}\left(2\times \pi \times {J}_{day}/{N}_{days}\right)$$

${S}_{ha}$ is the sunset hour angle, estimated as:

17 $${S}_{ha}= {\text{cos}}\left(-{\text{tan}}{(S}_{l}\right){\text{tan}}({S}_{d}))$$

${S}_{d}$ is the solar declination angle; ${J}_{day}$ is the Julian day of the year; and ${N}_{days}$ is the given number of days in a year.

Due to the unavailability of wind speed data in future timescales, its magnitude was considered as the average wind speed data during the baseline scenario. Consequently, all the estimated inputs were used to compute ${ET}_{0}$ using the FAO-56 PM approach and the season specific crop coefficient value at the respective locations of the command region were used assuming that the crop calendar remained unchanged as that of the baseline scenario to estimate the actual evapotranspiration (*ET*_*act*_). The SWAT-based comprehensive approach was adopted to obtain the water balance components of the concerned command region, and subsequently, the effective rainfall was estimated. The ${R}_{eff}$ of the concerned command region is computed from the SWAT water balance equation as given below^19^:

18 $${R}_{eff}=PR-{Q}_{s}-{Q}_{lat}-{Q}_{seep}$$

where ${R}_{eff}$ is the effective rainfall (mm); $R$ is the total rainfall touching the ground (mm); ${Q}_{s}$ is the streamflow (mm); ${Q}_{lat}$ is the loss in the form of lateral flow (mm); and ${Q}_{seep}$ is the water lost in the form of seepage (mm).

#### Environmental flow demand assessment

A range of environmental flow simulation approaches including the Hydrological Approach (HA)^29^, Hydraulic Approach (HUA)^30^, Habitat Simulation Approach (HSA), and Holistic Approach (HLA)^31^ are currently being operational across the globe. However, the HA approach, which explicitly depends on the historical streamflow time series is assumed to be the simplest yet effective approach and the HLA approach, which is a combination of hydrological information and biological factors is the complex most approach of E-flow estimation. However, unavailability in the large-scale habitat and corresponding biological factor information leads to non-adoption of the HLA approach more frequently. Hence, this study adopted the HA approach of E-flow estimation while computing the necessary demand components in the reservoir operation framework.

The conventional HA-based streamflow estimation approaches mostly rely on the analysis of monthly-scale streamflow time series and subsequent, 90^th^ percentile exceedance probability-based streamflow (referred as the Q_90_) is estimated and regraded as the Flow duration curve (FDC)-derived E-flow for the concerned catchment. However, this approach performed poorly under the hydrological noise scenarios prevalent during the monsoon and lean periods, resulting in erroneous E-flow estimation. To address this limitation, this study proposed a novel and reliable sub-monthly scale E-flow estimation approach. The suitable river sections having relatively stable flow velocity and water depth was identified over the Kangsabati river reach. The FDC analysis is approached on the observed daily streamflow data of the pre-identified reach under the pristine condition (Pre-dam construction period), i.e., 1950 to 1973, at a 10-daily scale. To reduce the relative noise during the monsoon/lean periods, each month was expressed as three sub-monthly periods (e.g., Jan-I, Jan-II, Jan-III), consisting of 10-daily averaged streamflow estimate. Further, each sub-monthly period streamflow time-series were subjected to Weibull’s plotting position analysis to perform the necessary FDC analysis and derive the Q_90_ value. Upon accumulating the estimated environmental flow for each sub-monthly period, the resulting monthly E-flow value was estimated. Subsequently, the net water demand was estimated as the sum of irrigation water requirement and E-flow requirement.

### Performance assessment of Kangsabati reservoir under future climate change scenario

The pre-calibrated and validated HEC-ResSim model was used to evaluate the developed operation rule in satisfying the demand of the command region in the future timescales. The reservoir inflow and SWAT estimated evaporation loss under different RCP scenarios as simulated by the SWAT model were fed to the HEC-ResSim model. Simulated reservoir release was compared with the demand for a given RCP scenario, and subsequently, the efficiency of the rule curve was evaluated.

As envisaged from the Kangsabati reservoir sedimentation survey (Table 1) by the Irrigation and Waterways Department, Government of West Bengal (GoWB), the Kangsabati reservoir faced acute sedimentation issue with a substantial reduction in the dead storage and live storage. With the assumption that the rate of reduction in the Kangsabati reservoir storage to be constant over the years, the average annual reduction in the reservoir live storage was estimated as 0.307%. Further, due to the reservoir restoration activity by the Kangsabati dam authority in the year 1994, its live storage was improved by 3% over a period of 30 years. Considering this rate would remain static in the future timescales, every 26-year span in the near-, mid-, and far- future period is expected to experience a reduction of 5.083% from the baseline period of 1970–1994. The reservoir simulation under the future RCP scenarios was carried out after updating the reservoir live storage values in the HEC-ResSim model.

**Table 1:** Reduction in Kangsabati reservoir storage capacity due to sedimentation (Irrigation and Waterways Department, GoWB).

Period	Capacity at the end of last survey period (m^3^)	Capacity at the end of given survey period (m^3^)	Cumulative loss (m^3^)	Cumulative loss (%)
1970–71	434,721,523.8	429,086,987.2	5,634,536.61	1.30
1976–77	429,086,987.2	423,822,494.5	10,899,045.52	2.51
1993–94	423,822,494.5	393,334,569.4	41,386,954.48	9.52

### Predictive uncertainty in reservoir performance under climate change scenario

The climate change impact analysis studies involving sustainable water resources management are subjected to multiple sources of uncertainty, including input data uncertainty, processing uncertainty, and modelling uncertainty. To ascertain the robustness of the proposed water resources planning framework, quantification of all the individual sources of uncertainty across the analysis time scale is quite inevitable. Hence, the uncertainty in the proposed future reservoir operation study could be attributed to the following sources, viz., the analysed emission scenarios, chosen GCMs, adopted downscaling scenario, and hydrological model. Certainly, the reservoir operation framework is dependent on the simulated reservoir inflow by SWAT model under multiple climate projections. Among the existing uncertainty quantification approaches, the present study adopted the maximum entropy (ME) based uncertainty quantification approaches as proposed by^32^ to individually analyse the inherent uncertainty in the precipitation, temperature, and simulated reservoir inflow as given below.

A given set of forcing variable characterized with maximum and minimum values of m and n, respectively, the corresponding maximum entropy (ME), i.e., H(x) is expressed as^33^:

19 $$H\left(X\right)=-{\int }_{m}^{n}{f}_{x}\left(x\right){\text{ln}}{f}_{x}\left(x\right) dx=-{\text{ln}}(n-m)$$

To qunatify the uncertainty involved with the streamflow projections, all the possible streamflow combinations (24 in this study) constituing the scenarios and models are generated. Then for model *j* corresponding to a given stage *i* the resulting maximum and minimum streamflow vaues are estimated and the corresponding maximum entropy, ME H_*ij* is_ estimated (Eq. 19). Further, individal stage specific maximum entropy values are identified with two selection options, viz., avaergae or maximum. However, the present study adopts the maximum selection approach to quantify the total uncertainty from various sources. The similar steps are repeated to quantify the uncertainty involved with the forcing variable upon excluding the source of uncertainty arising from the hydrological model SWAT.

## Study site and database

### Study area

**Figure 3:**
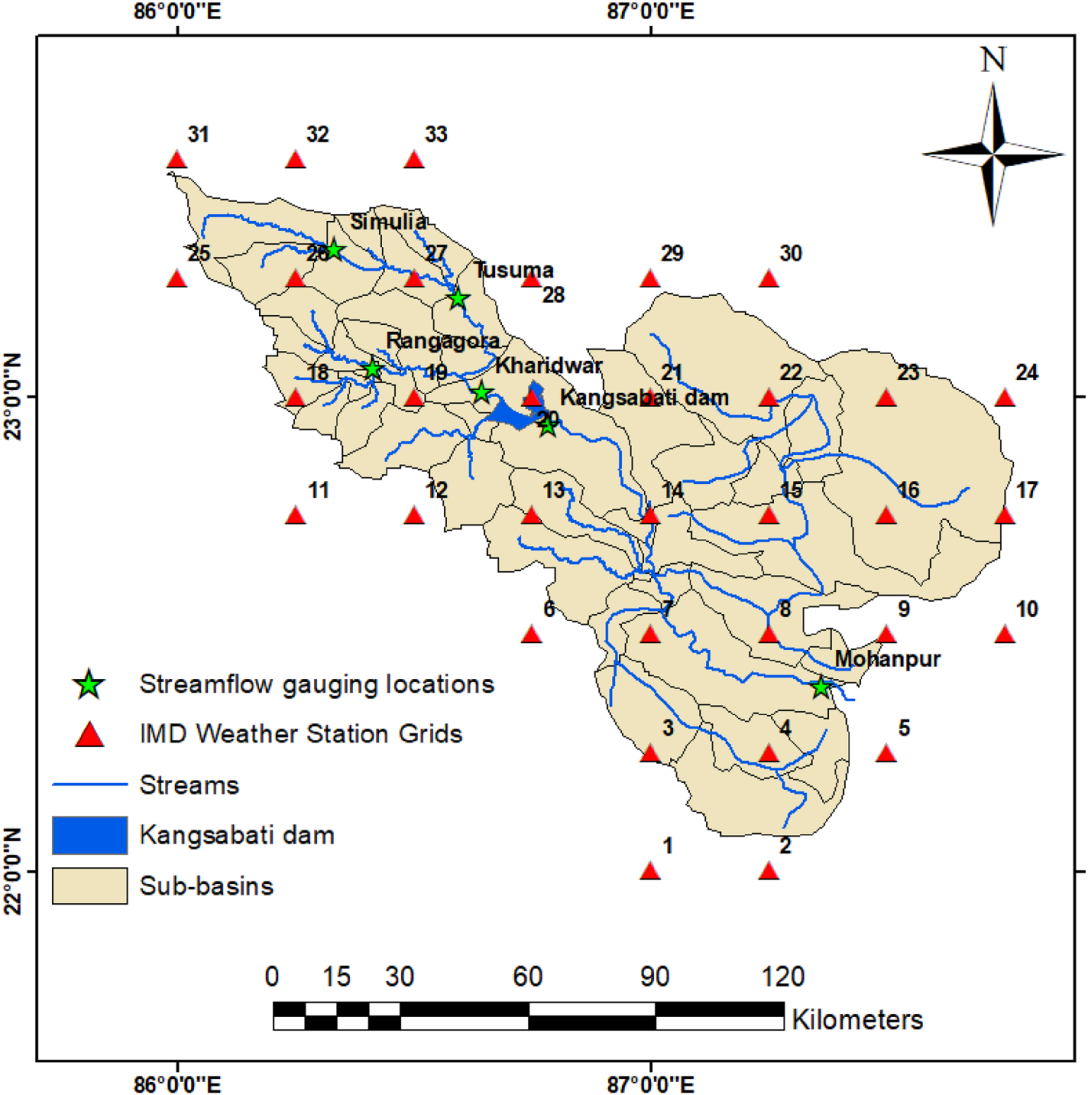
Index map showing river and reservoir catchment command locations.

The KRB is situated in the eastern part of India, which encompasses both reservoir command (5735 km^2^) and river catchment (6279 km^2^) regions (Fig. 3). The Kangsabati River starts in the Chhota Nagpur plateau, and subsequently, reaches the downstream location after covering a long flow path. The basin experiences highly undulated topography with the upper part of the basin characterized by high slope conditions. The study basin receives a mean precipitation of 1400 mm, of which 80% is concentrated during the monsoon period. The Kangsabati reservoir was built near the convergence point of Kangsabati and Kumari River. The dam was constructed in two phases with the first construction began in the year 1965. In the second phase, the dam construction over tributary Kumari was started in the year 1973, and subsequently, the Kangsabati reservoir was formed. The command area is encompassed between the districts of Bankura, Midnapur, and Hooghly of West Bengal having coordinate bounds of 22°08' to 23°13' N latitudes, and 86°45' to 87°47' E longitudes. The reservoir provisions the water flow through two main canal schemes, viz., Right Bank Main Canal (RBMC) and Left Bank Feeder Canal (LBFC) (Fig. 3).

The LBFC initially meets the Silabati river, where a barrage has been constructed, and subsequently, irrigates the area between the Silabati and Darakeswar river. The total length of the LBFC, i.e., from LBFC HR to SMC HR is approximately 9.9 km, whereas the length of KMC is about 21.1 km. The basic discretization of the Kangsabati canal system works in such a way that the main canal supplies water to the distributaries and to the direct outlet points, subsequently, the distributaries transmit water to the minors and sub-minors, whereas direct outlet points supply water to the field channels. Major agricultural crops are paddy during *kharif* season and wheat, potato and mustard are mostly grown during the *rabi* season. Summer (Boro) paddy is pre-dominantly cultivated in the command regions. The crop calendar of the k*harif* paddy is characterized by the sowing/transplanting period during mid-June to Early-July and harvesting is done during the month of October. In both LBFC and RBMC command area paddy growing regions combinedly constitute 48% of the total basin area.

### Pedo-hydrological database and pre-processing

The present study consisted of hydrological modelling of integrated river-reservoir catchment command; hence, the accuracy of this research is certainly pivoted around the availability of long-term and reliable input data. Observed forcing inputs, such as precipitation, maximum and minimum air temperatures, solar radiation, relative humidity, and wind speed at 1-daily temporal resolution available at six weather stations of Simulia, Kharidwar, Tusuma, Kangsabati dam, Rangagora, and Mohanpur for the period 1980 to 2011 were procured from the Central Water Commission (CWC), Asansol and India Meteorological Department (IMD), Pune.

**Figure 4:**
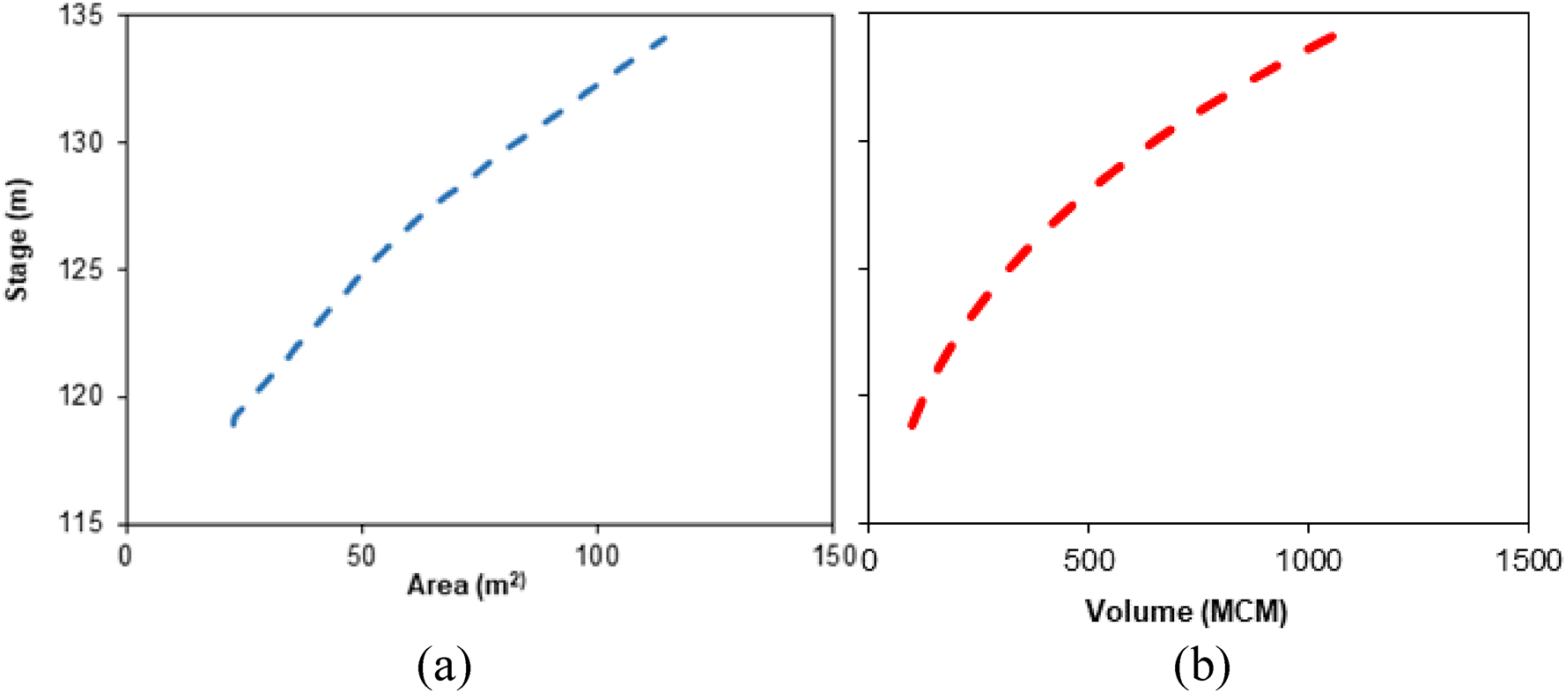
(**a**) Stage-area curve of Kangsabati reservoir; (**b**) stage-volume curve of Kangsabati reservoir.

The reservoir characteristic curves, viz., stage-area curve (Fig. 4a), stage-volume curve (Fig. 4b); dead storage, live storage, and daily flow releases at head regulators of LBFC and RBMC, downstream spillway discharge, daily reservoir inflow from the upstream catchment, and information about the canal network and their corresponding command regions for the period 1986–2011 were procured from the Office of the Superintending Engineer, Irrigation and Waterways Department, Bankura, Govt. of West Bengal. As per the information of the Water and Power Consultancy Services (India) Ltd. (WAPCOS, 2003), the field application efficiency was assumed to be 80% and the corresponding seepage loss in the canal networks is assumed to be 2.94 cumec/M sq. m.

Generally, the climate change impact assessment is carried out by forcing the GCM derived precipitation and temperature estimates to the previously calibrated and validated hydrological model. In this study, the CMIP5 derived five GCMs were chosen for projecting the future climatic conditions of the KRB under two RCP scenarios of 4.5 and 8.5.

## Results and discussion

### Evaluation of integrated hydrologic and reservoir simulation model performance

**Figure 5:**
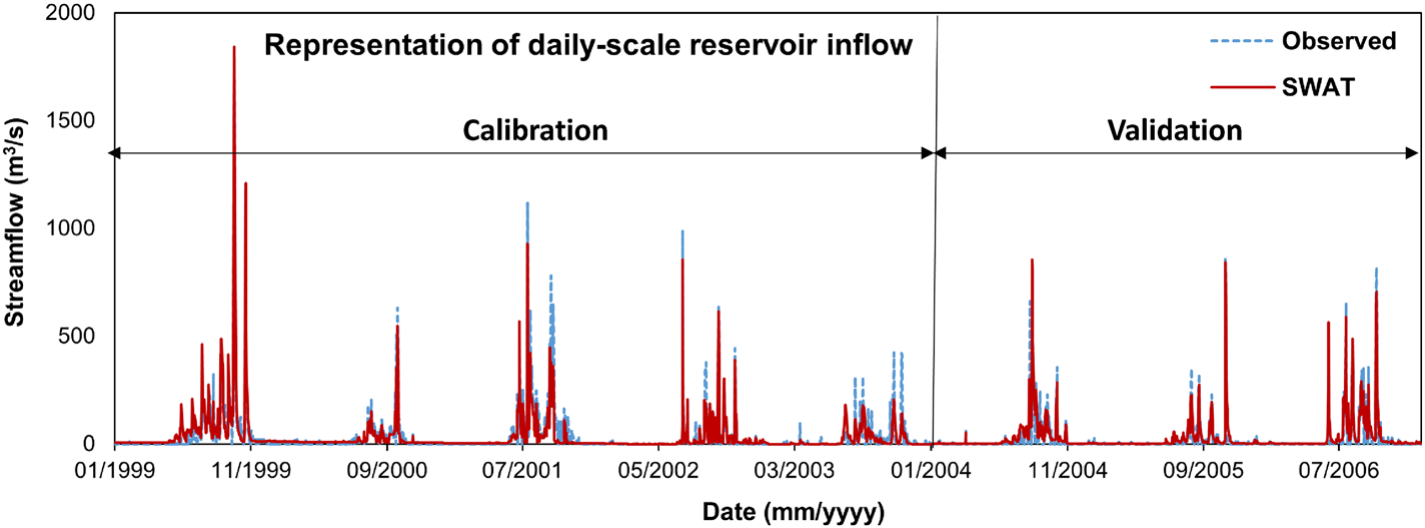
Representation of daily-scale reservoir inflow by the SWAT model.

The daily-scale representation of reservoir inflow during the calibration and validation periods is illustrated in Fig. 5, which revealed adequate representation of reservoir inflow during peak and lean water availability periods. As envisaged from Fig. 5, the daily-scale NSE and R^2^ ranged between 0.77–0.84 and 0.77–0.85, respectively during the calibration and validation periods. Considerable underestimation in the peak inflow is pre-dominant during the monsoon periods of the analysis horizon, which could be attributed to instantaneous high rainfall events. As depicted from RMSE estimates of 48.77 and 32.82 m^3^/s, respectively, the simulated inflow values are at par with the observed inflow estimates, and certainly, the chosen goodness of fit estimators are well within the acceptable limits as suggested by^34^. To identify the governing parameters of catchment-scale streamflow generation, an extensive sensitivity analysis was performed for the present study. Selection of hydrological model parameters plays vital role in governing the spatial simulation of the hydrological flux components. The model calibration was initiated with 26 commonly used parameters for streamflow simulation, and subsequently, using the Latin Hypercube One Factor at a Time (LHOAT) sensitivity analysis approach, 10 sensitive most parameters were included in the model calibration. The curve number (Fitted range of 47–86) turns out to be the top sensitive parameter, signifying that the reservoir upstream location surface water characteristic mostly governs the reservoir inflow simulation. The second most sensitive parameter, i.e., main channel conductivity (CH_K2) estimate of 30.7 mm/h depicts that the water travels from the upstream to the downstream section with moderate pace and maintains the time of concentration of the catchment. Moreover, few groundwater parameters including the soil hydraulic conductivity (SOL_K), baseflow alpha factor (ALPHA_BF), and deep percolation recharge (RCHRG_DP) are identified to be the sensitive parameters revealing the contribution of groundwater parameters to overall reservoir inflow simulation. Nevertheless, the HRU slope factor in the range of 0.2–14.32% plays a decisive role in rainfall-runoff translation.

**Figure 6:**
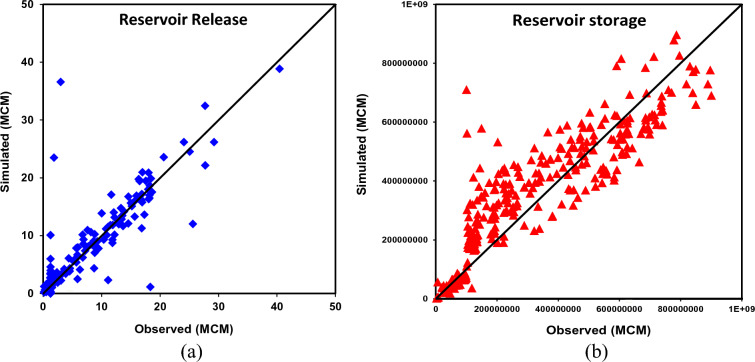
Reservoir simulation performance by the HEC-ResSim model: (**a**) reservoir release; (**b**) reservoir storage.

The monthly-scale reservoir release as obtained from the HEC-ResSim model alongside the observed inflow is illustrated in Fig. 6a, b. The reservoir release as simulated near the three outlets, i.e., LBFC, RBMC, and the main spillway are treated the combined outcome of the reservoir operation in the model conceptualization. The HEC-ResSim simulated reservoir outflow very well reproduced the observed counterparts across all the analysis time horizons; however, marginal mismatch could be noticed during the years 1989, 1993, and 2001. As depicted from goodness of fit indicators including NSE = 0.85, R^2^ = 0.89, and PBIAS =  + 1.23%, the HEC-ResSim model is quite effective in replicating the reservoir outflow pattern during both flood and drought phases. Nevertheless, the maximum reservoir release ranged between 20 and 31 MCM during the June–September months of the years 1989 and 1993, which were considerably underestimated from the simulated counterparts. Overall, with satisfactory performance at the canal network and their corresponding command regions both hydrological and reservoir simulation models, the proposed framework could be well adopted under both baseline and future climate change scenario.

### Integrated simulation–optimization based improved reservoir rule formulation

This study uses the simulated reservoir inflow and the estimated water demand as two primary inputs for the developed framework. Further, as per the proposed 10-daily E-flow estimation framework, the kharif season E-flow was estimated to be 126.37 MCM, and subsequently, added with the pre-estimated irrigation demand to compute the total water demand in the command region. The final fitted vales of the GA parameters including population size, mutation probability, and crossover probability were estimated to be 171, 0.01, and 0.72, respectively resulting in optimal system performance value of 8327 MCM with negligible deviation in the reservoir release is noticed.

**Figure 7:**
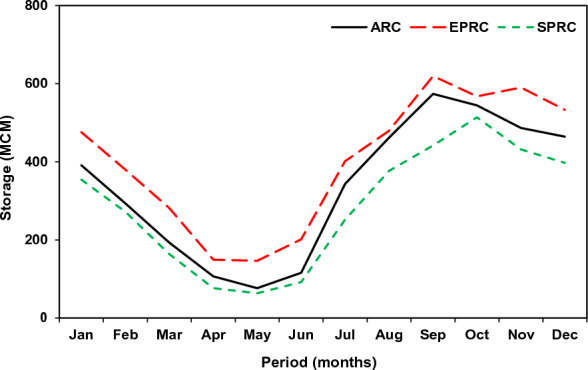
Derived reservoir operation rule curve for the baseline scenario.

The developed simulation–optimization framework is conceptualized by two representative priority scenarios, i.e., Supply Priority Rule Curve (SPRC) and Equal Priority Rule Curve (EPRC) scenarios, wherein the former corresponds to only reservoir release and the later refers to both reservoir release and flood control storage. The rule curve for the baseline scenario was constructed using the previously optimized GA parameters as presented in Fig. 7. As depicted from Fig. 7, both EPRC and SPRC based operation rules are swiveled around the original operation rule of the KRB; wherein significant deviation is observed during the post-monsoon period between the derived and the existing rule curve. The proposed rule curve revealed that the generated demand in the baseline scenario could be well met under the SPRC scenario, with relatively no scope for flood control storage. Owing to maximum release in the SPRC scenario, the storage level reaches a magnitude of 63.29 MCM during the month of May lead to critical dearth of future time horizon, which behaves as Standard Operating Policy (SOP) kind of reservoir operation. Conversely, the EPRC lowers the current demand of water marginally to contain flood storage and meet the plausible water demand in future like the hedge rule type operational procedure. Contemplating the applicability of EPRC in real world water management planning, the present study adopted the EPRC approach for both present and future analysis timescales.

Performance evaluation of the derived rule curve is analyzed under two major heads: first, actual water demand of LBFC and RBMC regions and second is practical water application scenario constituting 10–40% cut-off in the estimated water demand; henceforth termed as cut-off irrigation (CI) scenario. As more than 40% cut-off induces critical moisture stress in the crops, the cut-off in water demand is limited to 40% in the present study. Maximum volume reliability and time reliability estimates of 0.827 and 0.729, respectively, is depicted under the SPRC based operation scenario. Conversely, the previously described performance indicators attain their maximum value of 0.926 and 0.949, respectively under cut-off irrigation condition, depicting improved reservoir operation condition. Significant discrepancy is observed between the existing rule curve and EPRC derived rule curve as envisaged from the reservoir vulnerability and resiliency estimates of 0.127 and 0.671, respectively under 30% CI scenario. However, above 30% cut-off in actual irrigation obsoletes practicality in water resources planning; hence, the planning is limited to maximum 30% cut-off in the actual demand.

### Multi-model ensemble (MME) derived future climate change scenario

The magnitude of average annual rainfall as derived from the MME is in the decreasing order of MF (1733 mm) → FF (1611 mm) → NF (1529 mm); and MF (1689 mm) → FF (1623 mm) → NF (1569 mm) during the RCP 4.5 and 8.5 scenarios, respectively, with respect to the base period magnitude of 1395 mm at the upstream rain-fed location. This depicts that under both the RCP 4.5 and 8.5 scenarios, the MF period of the Kangsabati Reservoir is expected to face higher inflows that would cause adverse flood scenario in the downstream command regions. However, relatively higher monsoon rainfall could be expected in the command regions of the KRB yielding a positive impact on the crop growth during the lean periods. The projected temperature scenario in the KRB revealed that the MME-derived average monthly *T*_*mi*n_ reaches a value of 25 °C during the period of March–April-May under all the RCP scenarios with the least deviation among the individual GCMs. However, in the FF period, considerable difference is noticed among individual GCMs as envisaged from the standard deviation estimates of 3.02 °C and 2.27 °C for the reservoir and Mohanpur locations, respectively. Under the RCP 4.5 scenario, the T_max_/T_min_ temperatures are expected to increase in future as: NF (39.1/24.4 °C) → MF (40.6/25.4 °C) → FF (41.3/26.2 °C); while under the RCP 8.5 scenario, the T_max_/T_min_ temperatures are expected to increase as: NF (40.3/25.1 °C) → MF (41.4/26.3 °C) → FF (42.4/27.1 °C) depicting more intense heat wave scenario. As interpreted from the MME-derived projected temperature scenario at the upstream reservoir location, a relatively consistent projected temperature scenario could be expected in estimating T_min_ than the T_max_ with the highest T_max_ estimate of 44 °C to be observed in the FF period under the RCP 8.5 scenario.

Because of alteration in the future climatology in the study area, the water balance components are subjected to significant variation with respect to their magnitude in the baseline scenario. In this study, two major water balance components, i.e., streamflow and ET, depicting the supply–demand dynamics in the context of irrigation water demand are chosen for the analysis. The average annual trend estimates of streamflow and ET for the period 2020–2099 under three future time horizons of NF (2020–2045), MF (2046–2071), and FF (2072–2099) are presented in Tables 2 and 3. The estimated trends in the average seasonal future streamflow revealed a contrasting trend at the reservoir inflow location and the Mohanpur outlet. Similarly, there is a decreasing trend in the future streamflow only during the NF period at the reservoir inflow location, whereas in all the three-time horizons, the command area would experience a decreasing trend of streamflow. The considerable reduction in the streamflow could be attributed to the increased temperature projections over the KRB resulting in increased evapotranspiration loss. Conversely, the future ET projections over the KRB indicate an increasing trend by all the time horizons and RCP scenarios. However, the corresponding increase in the trend is more prevalent in the reservoir-command region than the upstream rain-fed regions as envisaged from the MK-test derived mean trend estimates of 1.987 and 2.788 in the MF and FF periods, respectively. Further, more intent ET loss is observed under the RCP 8.5 scenario, which could be due to the explicit interrelation between the ET and temperature variable.

**Table 2:** Seasonal streamflow projections derived from MME under RCP 4.5 and 8.5 scenarios.

Season	Reservoir inflow (m^3^/s)						Mohanpur outlet (m^3^/s)					
	NF		MF		FF		NF		MF		FF	
	RCP 4.5	RCP 8.5	RCP 4.5	RCP 8.5	RCP 4.5	RCP 8.5	RCP 4.5	RCP 8.5	RCP 4.5	RCP 8.5	RCP 4.5	RCP 8.5
Pre-monsoon	1.96	3.15	2.19	3.67	3.06	4.17	6.56	7.75	5.84	8.77	5.17	10.09
Monsoon	63.32	84.23	74.92	93.56	103.93	101.75	143.43	166.22	132.89	181.22	128.98	197.47
Post-monsoon	20.31	28.96	24.47	29.56	29.19	34.79	38.79	41.86	33.32	48.23	30.71	52.49

**Table 3:** Seasonal ET projections derived from MME under RCP 4.5 and 8.5 scenarios.

Season	Upstream rain-fed region (mm)						Command region (mm)					
	NF		MF		FF		NF		MF		FF	
	RCP 4.5	RCP 8.5	RCP 4.5	RCP 8.5	RCP 4.5	RCP 8.5	RCP 4.5	RCP 8.5	RCP 4.5	RCP 8.5	RCP 4.5	RCP 8.5
Pre-monsoon	211.54	234.32	228.55	240.43	237.21	257.79	291.76	323.67	311.78	334.96	320.21	324.31
Monsoon	506.32	528.89	554.32	592.87	637.89	648.87	698.23	712.65	712.78	727.82	741.26	754.98
Post-monsoon	331.09	343.74	351.27	397.61	417.28	431.23	420.76	431.02	448.49	462.31	461.21	477.41

**Figure 8:**
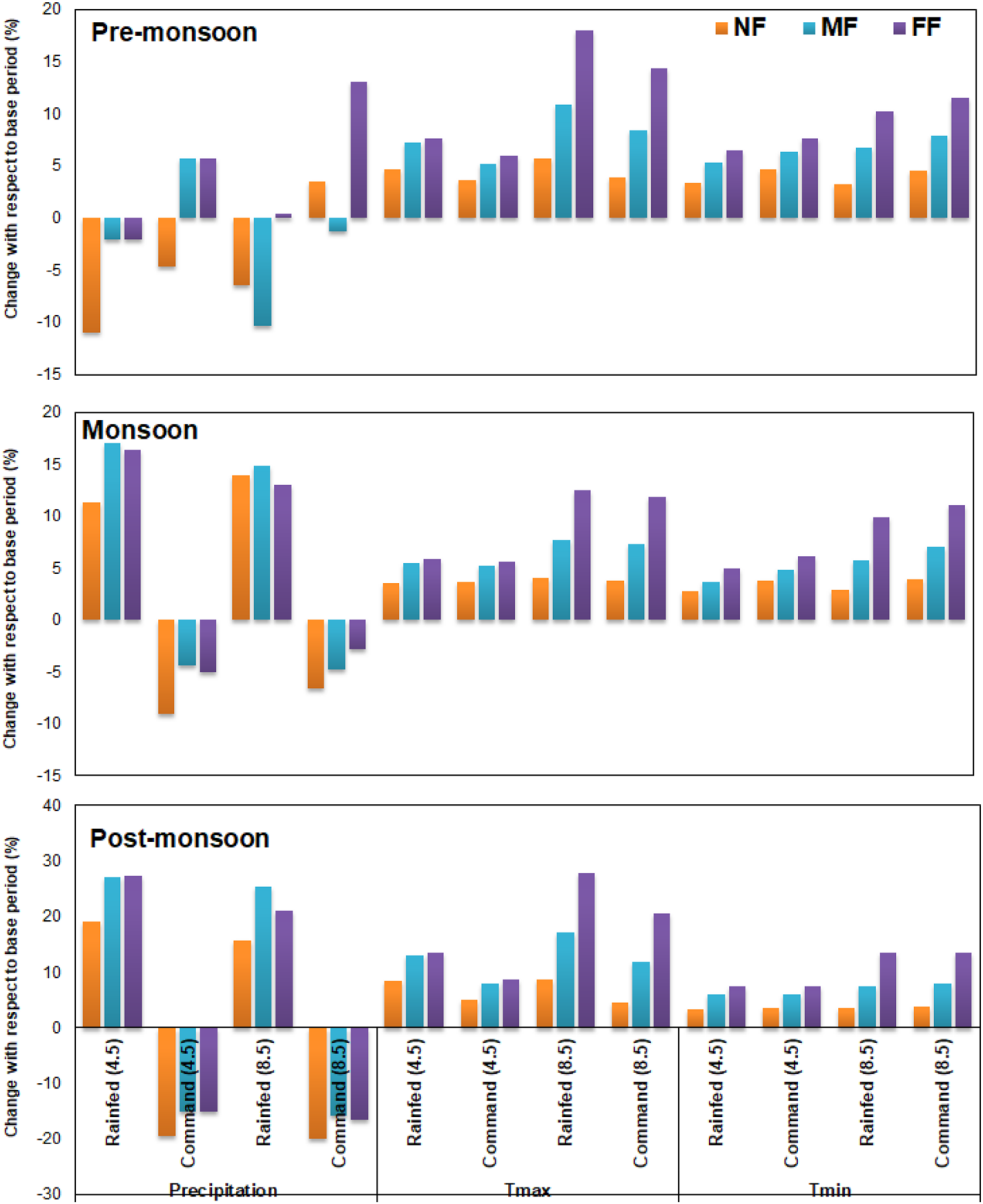
Mean seasonal precipitation change anomaly at the rain-fed and command regions in the study area for NF, MF, and FF periods with respect to the baseline (1951–2014) period under RCP 4.5 and RCP 8.5 scenarios.

**Figure 9:**
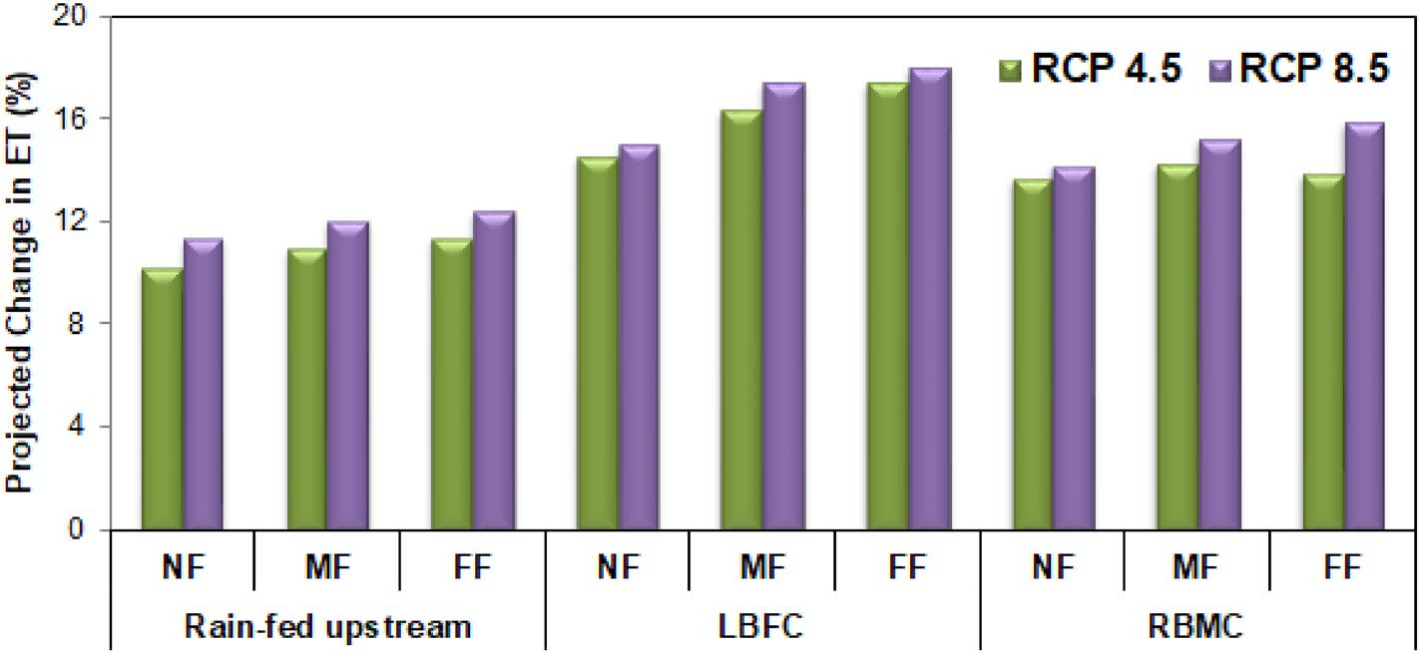
Relative increase in ET projections with respect to the baseline scenario at different test locations of KRB.

Similarly, the future ET projections revealed that these estimates are the highest during the month of August and the lowest during the month of February in the command region under both the RCP scenarios. The GCM derived future ET estimates are found to be in synchronous with the T_max_ and T_min_ variability. The future *ET*_*act*_ estimates are relatively higher under the RCP 8.5 scenarios by 13.14% and 17.32% from that under the RCP 4.5 scenario in the rain fed upstream and reservoir command regions, respectively. However, the monsoon period of the command region is likely to experience the highest increase in ET from the NF to FF periods. A detailed overview of the crop water requirement at the three test locations, viz., upstream rain-fed region, LBFC, and RBMC, the relative change in the ET magnitude with respect to the baseline scenario (1951–2014) is illustrated in Fig. 8. The MME derived *ET*_*act*_ projections in Fig. 9 revealed that the FF time horizon of the LBFC region may experience the highest increase in ET estimates of 18.02%. Corresponding the baseline scenario ET estimates of 1073 and 1297 mm in the rain-fed and command regions, respectively, the mean annual ET_act_ magnitude in the rain-fed and command regions are estimated to be 1158 mm/1226 mm and 1468 mm/1517 mm under the RCP 4.5/8.5 scenarios, respectively. This provides a clear insight into the increased water demands in the study area during the future time periods.

### Projected future irrigation water demand

**Figure 10:**
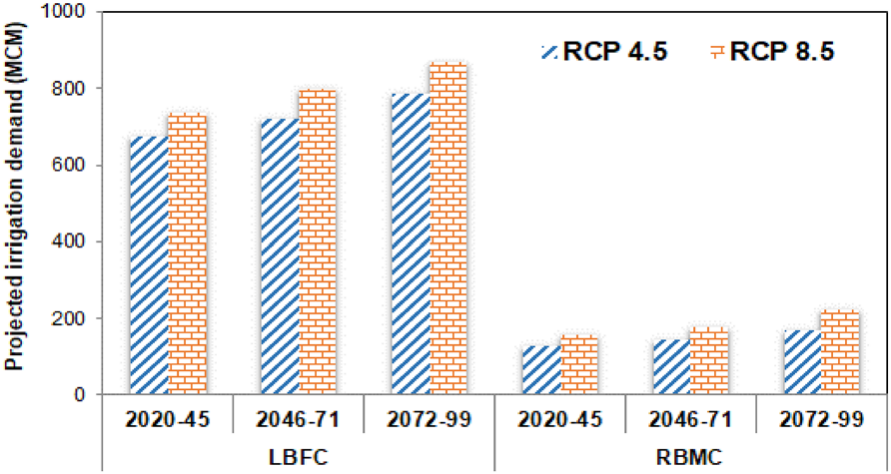
Future projected irrigation demand in the KRB command regions.

Since the change in E-flow requirement was assumed to be insignificant in the future scenario, the projected irrigation demand was added with the estimated E-flow in the baseline scenario to compute the projected net water demand. The mean projected irrigation demand during the *kharif* season at the LBFC and RBMC command is illustrated in Fig. 10. At both the command locations, the increase in irrigation demand under the RCP 8.5 scenario is relatively higher than the RCP 4.5 scenario, depicting the role of substantial decrease in rainfall in the command regions which may lead to reduced availability of effective rainfall for plant water uptake. The projected volumetric irrigation demand was the highest during the FF time periods under the RCP 8.5 scenario for both the LBFC (867.49 MCM) and RBMC (224.43 MCM) command regions. Moreover, the intent increase in the projected ET during the NF, MF, and FF periods seems to have direct influence on the irrigation water requirement scenario, wherein the projected irrigation water demand at both the LBFC and RBMC are reduced in the order of FF → MF → NF. Although both the rainfall and temperature variables are subjected to increase in the future scenario, the relative impact of ET is higher in governing the future irrigation demand. Moreover, the mean heatwave intensity of 2.84 °C during the FF period under the RCP 8.5 scenario with prolonged event duration of 10–12 days could have caused substantial moisture stress in the command region aggravating the irrigation water demand to a greater extent under the RCP 8.5 scenario. Finally, the estimated irrigation demand and previously estimated E-flow requirement are combined to estimate the total water demand in the future scenarios, and subsequently, used in the reservoir simulation–optimization model framework.

### Assessment of reservoir performance under future climate change scenario

To facilitate the climate change impact on reservoir operation, the SWAT model was used to simulate the Kangsabati reservoir inflow for the NF, MF and FF periods under two RCP scenarios of 4.5 and 8.5. The reservoir performance under the future climate scenario was assessed by comparing the simulated outflow and estimated future demand as indicated by the time reliability, volume reliability, resiliency, and vulnerability indices. Under unsatisfactory reservoir performance condition, necessary modification to the existing operation rules was applied. The SWAT simulated reservoir inflow and estimated water demand for the RCP 8.5 scenario was adopted in the previously proposed SWAT-HEC-ResSim-GA framework to derive the optimized rule curve for the RCP 8.5 scenario. The applicability of the EPRC-derived reservoir operation rule pertaining to the actual future water demand as simulated under the MME-derived future climate change scenarios are presented in Table. 4.

**Table 4:** Performance of Kangsabati reservoir during future timescales of RCP 4.5 and RCP 8.5 scenarios.

Performance index	RCP 4.5			RCP 8.5		
	NF	MF	FF	NF	MF	FF
Time reliability	0.621	0.584	0.611	0.512	0.389	0.402
Volume reliability	0.657	0.593	0.638	0.595	0.456	0.491
Resiliency	0.547	0.497	0.512	0.518	0.342	0.473
Vulnerability	0.203	0.226	0.217	0.213	0.345	0.306

**Figure 11:**
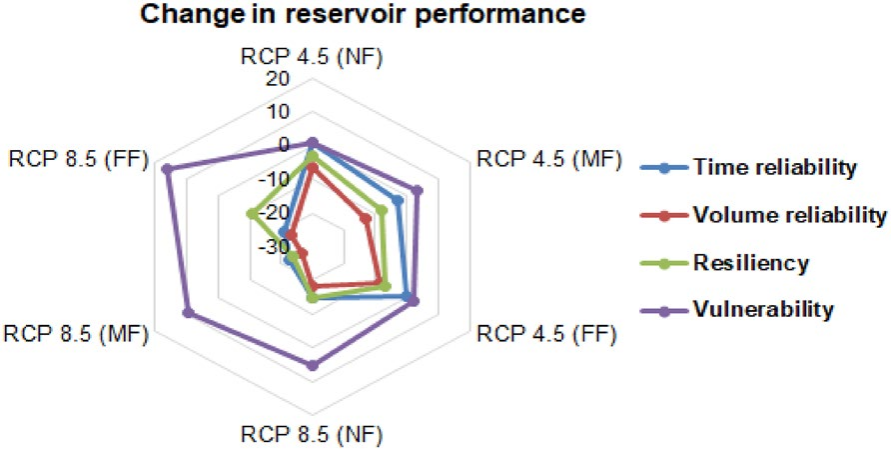
Kangsabati reservoir performance under the future climate change scenario.

It is evident from Table 4 that the efficiency in reservoir operation under NF period gradually degrades during the MF and FF periods under the two RCP scenarios. The SWAT-HEC-ResSim-GA derived reservoir operation rules for the baseline scenario revealed that the reservoir release could satisfactorily meet the desired water demands during all the NF, MF, and FF episodes under the RCP4.5 scenario with the reservoir time reliability estimates of 0.621, 0.584, and 0.611, respectively; volume reliability of 0.657, 0.593, and 0.638, respectively; resiliency of 0.547, 0.497, and 0.512, respectively; and vulnerability of 0.203, 0.226, and 0.217, respectively. However, during the MF/FF episodes of the RCP 8.5 scenario, the Kangsabati reservoir is projected to perform poorly with reduced time and volume reliability estimates of 0.389/0.402 and 0.456/0.491, respectively, which can be attributed to the 19% and 22% increase in the projected irrigation water demands during the MF and FF episodes from the baseline scenario. This necessitates a clear understanding of how the individual reservoir performance evaluation index varies corresponding to the baseline scenario. To ascertain the relative lowering in future reservoir operation performance with respect to the baseline scenario, the percentage change in the individual performance index corresponding to its counterpart in the baseline scenario is presented in Fig. 11.

As seen from Fig. 9, the reliability, resiliency, and vulnerability estimate for the Kangsabati reservoir have significant variability with respect to the baseline scenario (1986–2011). Across all the future time-horizons, the RCP 4.5 scenario is subjected to minimal alteration in the range of <  ± 10% for the four reservoir performance indices. The minimal reduction in the reservoir performance under the RCP 4.5 scenario signifies the improved applicability of the proposed operation rule under baseline scenario. Conversely, the RCP 8.5 scenario corresponds to much adverse water supply–demand scenario in the future timescales with significantly varying reservoir performance index estimates from that of the baseline scenario (1986–2011). Among the four performance evaluation criteria considered herein, the vulnerability criterion under the RCP 8.5 scenario for the far-future period is found to be the highest (34.5%) than that of the RCP 4.5 scenario (22.6%). Overall, the proposed reservoir operation rule for the baseline scenario performed satisfactorily under RCP 4.5 scenario. Moreover, the MF and FF periods under RCP 8.5 are likely to experience significant reduction in the reservoir volume reliability and resiliency indices in the range of 23.1–2.6 and 10.6–23.7%, respectively, revealing the necessity to modify the proposed operation rule under baseline scenario. To address this limitation in the reservoir operation rule under the RCP 8.5 scenario, the SWAT-HEC-ResSim simulated rule curve was updated by the GA-based optimization technique in which the projected future water demands and reservoir inflows for the RCP 8.5 scenario were used as the input variables.

**Figure 12:**
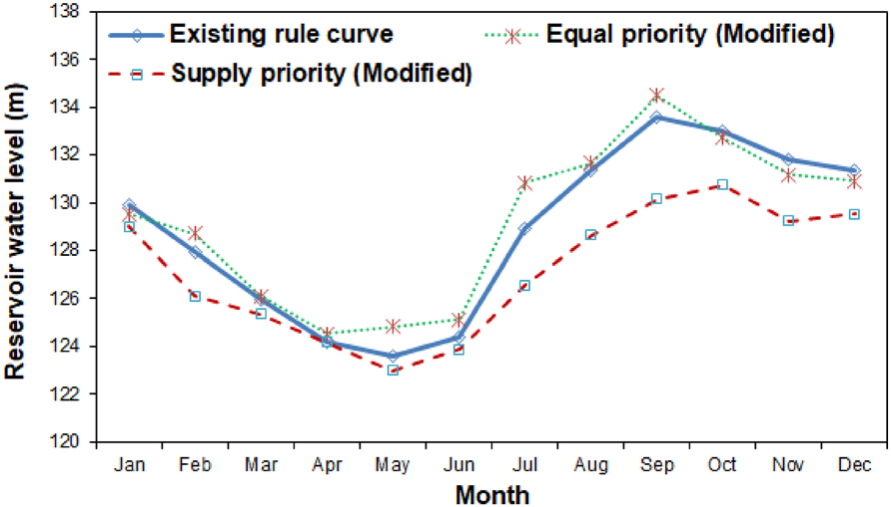
Proposed optimized rule curve for the Kangsabati reservoir under the RCP 8.5 scenario.

As can be seen from Fig. 12, the optimized revised rule curve so generated under the RCP 8.5 scenario could ensure efficient irrigation release with time and volume reliability estimates of 0.623 and 0.651 for the equal priority rule curve scenario, respectively. The revised reservoir rule curve under the RCP 8.5 scenario revealed that the simulated reservoir releases during the period of April-August are increased by 3–7% under the RCP 8.5 scenario; while during the lean periods (January-May), the simulated release are increased by approximately 12% to address the acute water demands in the downstream command area under the equal priority scenario. The revised operation rule under the RCP 8.5 scenario derived by the SWAT-HEC-ResSim-GA framework resulted in reduced numbers of irrigation failures from 64 to 38 and from 43 to 29 during the MF and FF periods when compared with the baseline period. This caused significant improvement in the reservoir performance under the RCP 8.5 scenario with the time/volume reliability estimates of 0.596/0.607 and 0.623/0.651 during the FF and MF episodes, respectively, as compared to the baseline period derived rule curve with the corresponding estimates of 0.402/0.389 and 0.491/0.456 in the FF and MF episodes. Consequently, the reservoir resiliency and vulnerability also improved to 0.581/0.502 and 0.223/0.241 in the FF and MF episodes, respectively.

**Figure 13:**
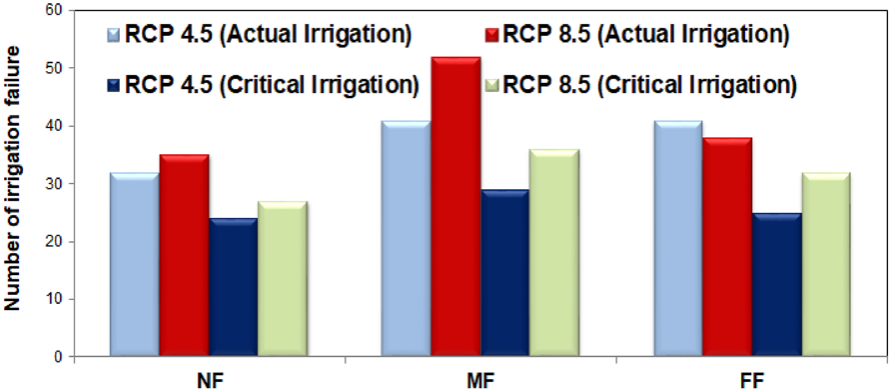
Projected number of irrigation failures under the RCP 4.5 and 8.5 scenarios for the near-future (NF), mid-future (MF) and far-future (FF) time episodes.

Further, adopting the revised rule curve under the RCP 4.5 scenario also resulted in substantial improvement in the reservoir performance as envisaged from enhanced reservoir performance indices (time reliability/volume reliability/resiliency/vulnerability) of 0.639/0.671/0.563/197, 0.598/0.613/0.514/0.201, and 0.632/0.669/0.532/0.194 during the NF, MF, and FF episodes, respectively. Although the supply priority derived rule curve ensures higher reliability and resiliency value, the reservoir releases during most of the monsoon periods exceeds the maximum channel carrying capacity leading to probable flood scenario in the downstream. Hence, the equal priority-based rule curve also seems to be the best possible reservoir operation scheme under the RCP 4.5 scenario. The number of times the simulated reservoir release fails to meet the desired water demand is treated as irrigation failure and becomes the true representative of the efficiency of the proposed reservoir operation rule. The number of irrigation failures as simulated by the SWAT-HEC-ResSim-GA conceptualization using the modified rule curves proposed for the RCP 8.5 scenarios with the actual and critical irrigation demands is presented below in Fig. 13. As envisaged from Fig. 13, the number of irrigation failures will be reduced substantially in the future time scales from that of the existing rule curve-based operation in the baseline scenario. Considering the actual irrigation demand, the number of failures is estimated to be relatively lower under the RCP 4.5 scenario; however, considering the pre-assumed 30% curtailment in the actual demand, the number of failures is reduced by 19.72% in the RCP 8.5 scenario corresponding to the baseline proposed rule curve release scenario. This depicts that the magnitude of irrigation demand is projected to be higher under the RCP 8.5 scenario as compared to the RCP 4.5 scenario for the MF and FF periods. By adopting the proposed revised reservoir rule curve under the RCP 8.5 scenario, the average reduction in the number of irrigation failures, pertaining to the 30% cut-off in the actual irrigation demand, is estimated to be 31.10% and 23.13% under the RCP 4.5 and RCP 8.5 scenarios, respectively. This depicts that under critical water availability scenario, with 30% cut-off in the actual water demand, for instance, the Kangsabati reservoir would perform more efficiently under both the RCP scenarios. The number of irrigation failures presented in Fig. 13 revealed that the proposed SWAT-HEC-ResSim-GA simulation–optimization based reservoir operation framework could perform better in the reservoir operation under climate change scenarios. Therefore, development of similar improved adaptive reservoir operation rules under future climate change scenarios has the potential to aid the decision makers in formulating sustainable operation policy for other multi-purpose reservoirs worldwide. Conclusively, the reservoir performance assessment scheme implemented herein could effectively address the possible additional demand sectors like inter-basin water transfer and domestic water requirement in the catchment-scale applications.

### Assessment of uncertainty in reservoir performance

As envisaged from Table 5, the cumulative uncertainty in streamflow simulation goes on increasing with the advancement of different processing stages. The final cumulative uncertainty as obtained in the end of Table 5 represents the total uncertainty estimate of 5.26. Certainly, the uncertainty contribution to the streamflow simulation was the highest from analysis scenarios (55.90%) followed by GCMs, downscaling technique, and chosen hydrological model. Further, more uncertainty is arising from the RCP 8.5 scenario with ME estimates of 2.94 corresponding to the RCP 4.5 scenario (ME = 2.12), signifying that the streamflow projections obtained in this study are more reliable in the RCP 4.5 scenario. A close observation at the relative contribution to uncertainty in streamflow simulation by the individual GCM and the MME revealed that the MME derived streamflow simulation yielded the lowest uncertainty in long-term future streamflow simulation with ME estimate of 3.31 corresponding to the high ME value of 4.91 in case of the MIROC-ESM-CHEM model. These findings are in line with the recommendation of many previous studies that the MME derived climatic projections give more reliable estimation of the future streamflow with least uncertainty^35,36^. Moreover, the natural variability corresponding to the uncertainty in the baseline scenario having magnitude 56.46% is quite comparable with the future streamflow projections with less than 50% increased uncertainty. Hence, the projected streamflow simulations obtained in this study are accurate enough in deriving the future reservoir operation rule for both the RCP 4.5 and 8.5 projections with slightly higher degree of overall uncertainty in the RCP 8.5 scenario.

**Table 5:** Quantification of reservoir inflow simulation uncertainty under climate change scenario.

Analysis stage		Maximum entropy (ME)	Maximum of ME	Incremental ME	Uncertainty ratio (%)
RCP scenarios	RCP 4.5	2.12	2.94	–	55.9
	RCP 8.5	2.94			
GCMs	BCC-CSM1-1	3.98	4.91	1.97	37.45
	GFDL-ESM2G	4.33			
	MIROC5	4.87			
	MIROC-ESM-CHEM	4.91			
	NorESM1-M	4.29			
	Multi model ensemble (MME)	3.31			
Downscaling technique	MLR	3.82	5.17	0.26	4.94
	KR	5.17			
Hydrological model	SWAT	5.26		0.09	1.71
Total uncertainty		5.26	5.26	–	100
Natural variability		2.97	2.97	–	56.46

**Figure 14:**
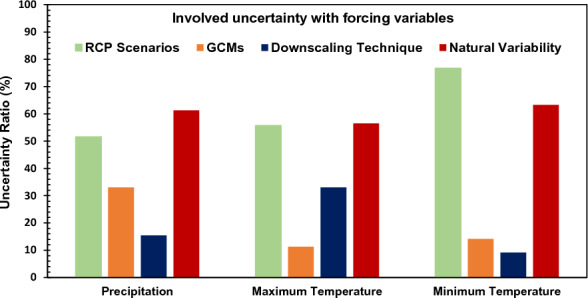
Relative uncertainty in the projected forcing variables for the future scenario.

To ascertain the uncertainty involved with the projected forcing variables, Fig. 14 revealed the relative uncertainty contribution from different RCP scenarios, GCMs, and adopted downscaling technique. Like the streamflow, the RCP scenarios contributed the highest uncertainty for all the three forcing variables, the highest uncertainty being observed for the minimum temperature variable (76.68%). However, the adopted downscaling technique contributed to least uncertainty (< 20%) depicting better special discretization of all the forcing variables for the future simulation. As expected, the RCP 8.5 scenarios corresponded to the highest overall uncertainty in case of all the derived future forcing variable projections. Overall, the uncertainty assessment performed herein revealed that the future projected uncertainty is 43.54% higher than the baseline scenario and the proposed reservoir operation rules can be adopted in future decision-making studies with reasonable accuracy.

## Discussions

Broadly, the reservoir release operation is described under two primary modelling heads, viz., open loop reservoir model and close loop reservoir model. The open loop model exclusively relies on historical hydro-meteorological data and pre-defined rule curves for governing the reservoir release. Considering relatively simpler model conceptualization and higher computational efficiency, the open loop-based reservoir simulation models were extensively used in many past studies^37–39^. Conversely, the closed loop model which accounts for the real time hydrological condition and demands to adjust the release decision dynamically. However, excessive computational requirement and required access to real-time hydrological information constrains the application of closed loop reservoir models^40^. Moreover, the application of the closed loop reservoir models under climate change scenario may yield unreliable performance because of increased uncertainty form the climate model and machine learning or data assimilation approaches. The proposed integrated reservoir simulation strategy, which adopts both the hydrological and reservoir simulation approach in conjunction with the dynamic environmental flow and demand estimation approach can be treated as a semi-closed reservoir modelling approach with satisfactory performance across both historical and future climate change scenarios.

The future water demand projection is in line with the outcomes form past support vector regression-based demand estimation study performed over the Netherland and Belgium region, depicting 0.8% increase in the demand by the period 2050^41^. Owing to the high temperature and varying precipitation behaviour, the temperate region may face more severe water scarcity issue than the tropical region. A study on the global future water demand revealed that few parts of India and Middle East may experience water demand from the hydroelectricity sector, although more emphasis on import of food products will reduce the agricultural water demand to some extent^42^. Further the increased future water demand in the concerned region could be attributed to more paddy cultivation, which is evident in the study conducted by^43^, depicting that intense cereal and paddy cultivation may lead to increased water demand under baseline and future climate change scenario.

An approach by^37^ accounted the synergistic relationship between the environmental flow and the cropping pattern optimization conceptualization under climate change scenario, revealing that maintaining both irrigation demand and environmental flow might be challenging under low reservoir inflow condition. In support of the findings by^37,44^, the present study depicted that under adequate precipitation and reservoir inflow scenario, the proposed reservoir operation rule can meet both irrigation demand and environmental flow adequately, highlighting the explicit climatic control on the supply–demand dynamics. An overview on the climate change impact on the reservoir operation revealed the difference between the supply–demand was the highest during the dry periods of many global studies, which is also evident in the current study region during the month of April and May^45–47^. To ascertain more practical water management scenario, 30% cut-off in the actual irrigation demand is recommended in this study, which is in line with the study conducted by^48^ depicting 30% reduction in agricultural demand under RCP 4.5 scenario resulted higher sustainability index.

The proposed reservoir operation framework integrated the hydrological modelling framework with the reservoir simulation framework to evaluate the efficiency of the derived rule curve. The conventional hydrological models like SWAT and VIC adopted extensively in reservoir operation studies do not represent the actual stream-reservoir connectivity; hence the modelling outcomes are less reliable^49,50^. Moreover, the HEC-ResSim based reservoir simulation model provides the option to define the reservoir operation rules while evaluating the reservoir operation performance across the concerned river basin. A comparative study between the Soil Conservation Services-Curve Number (SCS-CN) method and Artificial Neural Network (ANN) for Kangsabati reservoir inflow simulation revealed poor performance owing to the involved complexity of catchment and reservoir^51^. Many past studies tend to ignore the primary loss component, viz., evaporation loss while estimating the overall water balance, resulting in an erroneous assessment of demand components^52^. Assessment of environmental flow under limited data availability conditions hinders the adoption of habitat-simulation and holistic approaches^8^. In this context, the hydrological analysis based FDC approach is extensively adopted in past studies; however, all the previous studies performed the analysis in a monthly-time scale depicting significant uncertainty^53^. To account for the underlying uncertainty, the proposed novel sub-monthly scale E-flow estimation technique resulting in more realistic E-flow quantification for the reservoir operation; thereby improving the overall demand assessment of the study catchment. Certainly, the improved E-flow estimation approach can enrich the decision makers with more confidence in assessing the downstream E-flow requirement.

Lack of suitable adaptive operating policy results in reduced reservoir performance along the future climate change scenarios. As envisaged from a study on the Descoberto reservoir, the reservoir reliability reduced in the range of 15–50% during future climate scenarios of 2031–2080 from the initial value of 100% during the baseline scenario, pointing towards the risk of water management under highly non-stationary condition^54^. The developed approach analyzed the reservoir performance under a range of irrigation application and water demand scenario to determine the best possible adaptation option for maintaining the reservoir performance at optimal level. Certainly, the 30% cut-off irrigation strategy suggested in this study can maintain the reservoir reliability to more than 95% from its corresponding value in the baseline scenario. The developed approach proved to be an advancement over the previous reservoir operation studies, wherein the reservoir performance reduced drastically in the climate change scenarios. The proposed hydrological modelling-based reservoir inflow and hydrologic flux estimation strategy effectively conceptualized the catchment dynamics and undergoing management operations; thereby addressing the limitations raised in the previous studies^51^. The primary concern of the non-availability of long-term hydrological and environmental conditions of pre and post dam construction period fails to mimic the true hydrological behaviour^55^. In many past studies, with the stationarity assumption, the past hydro-meteorological datasets are extrapolated, giving incomplete estimate of future water availability scenario^56^. The integrated proposed approach effectively simulates the reservoir water balance and catchment hydrological behaviour to effectively predict the future supply–demand scenario. The developed HEC-ResSim reservoir simulation model accounts for detailed hydraulic components of the river-reservoir system and all the involved demand components in the conceptualization to design the operation rule curve with utmost accuracy. Moreover, the proposed 10-daily scale E-flow assessment has the potential to quantify the corresponding demand component with lower uncertainty than the existing monthly E-flow assessment approaches. The integrated methodology is subjected to inherent predictive uncertainty arising from model, climate scenario, and input data, which is ignored or estimated as a single component in most of the previous studies^57^. The uncertainty quantified herein adds more confidence to the overall assessment framework by segregating the relative uncertainty from different sources, thereby improving robustness. Given these improvements, the scenario-based reservoir operation framework proposed in this study is a novel approach on its own; adding substantial flexibility to derive the reservoir operation rule by giving complete priority to irrigation demand in agriculture dominated catchment and varying weights under mixed demand conditions. The proposed integrated methodology is in the form of a loose coupling; hence, simultaneous update of the simulated reservoir inflow, reservoir outflow, and storage can be achieved through a tight coupling of the SWAT and HEC-ResSim models. The upcoming studies may integrate a hybrid machine learning framework with the developed approach for real time and near future performance assessment of the reservoir system with utmost accuracy.

The proposed approach certainly behaves as a suit of decision-making tool having potential to estimate the supply and demand components under both baseline and climate change scenario. The policy makers can adopt this approach with high reliability to ascertain ubiquitous reservoir operation policy under highly non-stationary future climate change scenario. Moreover, provision of futuristic correction and practical irrigation planning proposed herein will enrich the policy makers with more realistic crop planning and integrated water management.

## Conclusions

Some of the key findings from this present study are given below:

i. The developed SWAT-HEC-ResSim-GA based simulation–optimization framework could effectively address the irrigation water demand and flood control objectives under the ‘equal priority’ rule curve scenario with the satisfactory time and volume reliability estimates of 0.614 and 0.722, respectively. Although the ‘supply priority’ rule curve scenario provides more reliability and resiliency than that of the ‘equal priority’ rule curve scenario, with a reduced reservoir storage of 50.21 Mm^3^ and increased irrigation failure of 37 during the period June–December against 16 irrigation failures during January–May, the reservoir operation behaves more like a standard operating policy.
ii. The GCM-derived future climate change scenario revealed that the mid-future period would be the extreme rainfall projections during both RCP 4.5 (1733 mm) and RCP 8.5 (1689 mm) scenarios with declined projections are confined to the pre-monsoon season. Similarly, the future temperature projections revealed that the upstream rain-fed locations would be the hottest during the RCP 8.5 scenario with mean annual *T*_*max*_ estimate of 43ºC. However, the degree of disagreement between the individual GCM projections gradually increased from RCP 4.5 to RCP 8.5 scenario.
iii. The MK-test and Sen’s slope trend statistics of the two major water balance components indicated a decreasing trend for the streamflow (only during NF) and increasing trend for the ET across both the rain-fed upstream and downstream command locations. However, significant trend for ET is observed during the RCP 8.5 period at the KRB command regions. The declined trend of streamflow at the Kangsabati reservoir inflow location would result in reduced projected reservoir storage.
iv. The inter-seasonal variability of streamflow was the highest during the monsoon season. Moreover, the LBFC is projected to be more drought-prone as compared to the RBMC and upland rain-fed locations with 18.02% increase in the mean ET loss during 2020–2099 with respect to the baseline scenario.
v. The proposed rule curve for the baseline scenario could satisfactorily meet the desired water demands during all the near-, mid-, and far-future episodes under the RCP4.5 scenario with the reservoir time reliability estimates of 0.621, 0.584, and 0.611, respectively; volume reliability of 0.657, 0.593, and 0.638, respectively; resiliency of 0.547, 0.497, and 0.512, respectively; and vulnerability of 0.203, 0.226, and 0.217, respectively.
vi. The proposed rule curve for the baseline scenario could not perform satisfactorily under RCP 8.5 scenario of mid- and far-future period with both time and volume reliability estimates of below 0.5. However, the SWAT-HEC-ResSim-GA based proposed revised operation rule under the RCP 8.5 scenario improved the Kangsabati reservoir performance in satisfying the irrigation demand with substantial reduction in the reservoir vulnerability estimates of 0.211 and 0.197 from that of the existing reservoir operation rule vulnerability estimates of 0.345 and 0.306 during the mid- and far-future periods, respectively.

The proposed SWAT-HEC-ResSim-GA framework proved to be an effective advancement over the conventional reservoir operation strategy with provision for futuristic update of hydrometeorological conditions, quantifying the underlying uncertainty arising from different phases of decision-making, novel environmental flow assessment approach, and capability to conceptualize all the supply/demand components in the modelling framework. To enhance the robustness and accuracy of the proposed approach, the follow-up studies may integrate a hybrid machine learning algorithm in the currently proposed framework to enable real time reservoir operation policy. The machine learning based real-time error updating scheme can address the plausible error arising due to the climate model uncertainty in the future climate change scenario. Conclusively, the developed framework can assist the decision makers in systematic planning of integrated river-reservoir catchments with increased confidence and can be well extended across any global catchment.

### Supplementary Information

Supplementary Information.

## Data Availability

The data that support the findings of this study are available from the Central Water Commission (CWC), Asansol, India, but restrictions apply to the availability of these data, which were used under license for the current study, and so are not publicly available. Data are however available from the authors upon reasonable request and with permission of the Central Water Commission (CWC), Asansol, India.
