# Supplementary material for: An integrated reservoir operation framework for enhanced water resources planning
Source: Sci Rep. 2023 Dec 8;13:21720. doi: 10.1038/s41598-023-49107-z (PMC10709336; doi:10.1038/s41598-023-49107-z)
Supplement: Supplementary file 1 — Supplementary Information. [file 41598_2023_49107_MOESM1_ESM.docx]

**List of Figures**


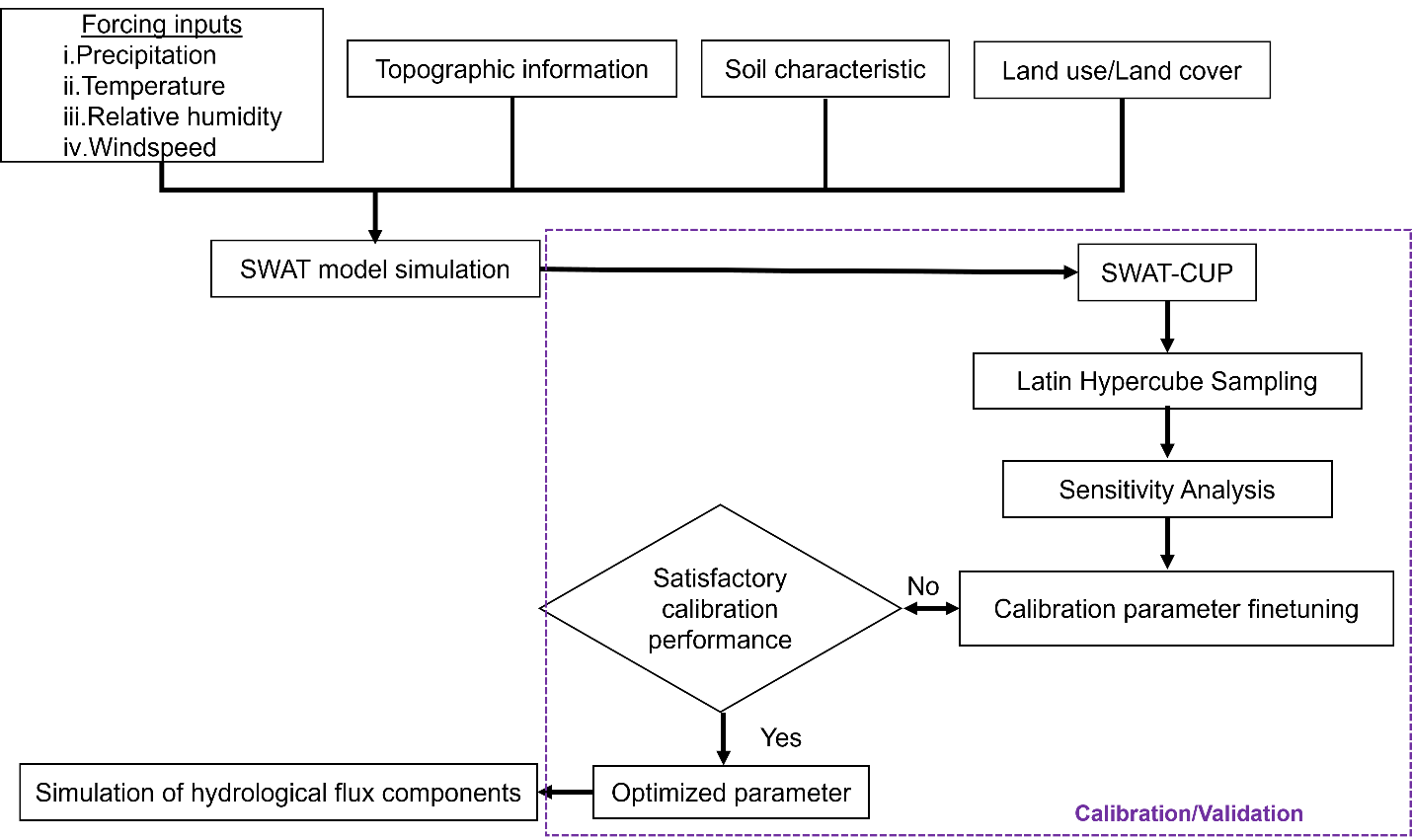


Fig. A1. Process flowchart for reservoir inflow simulation by the SWAT model.


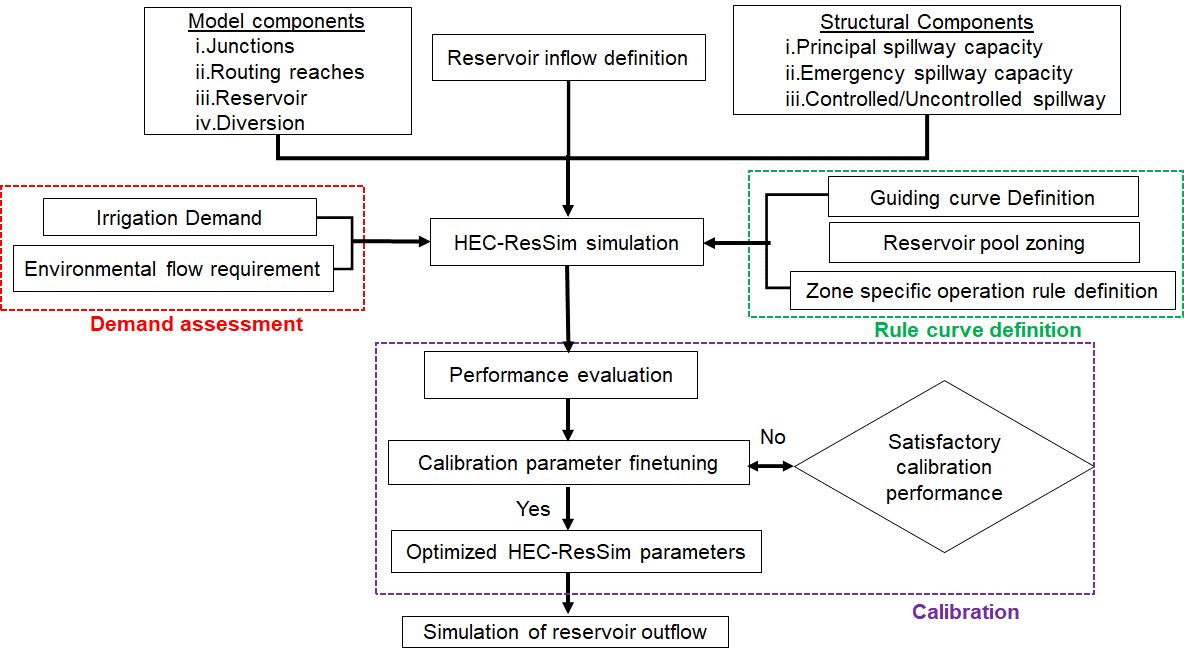


Fig. A2. Process flowchart for reservoir outflow simulation by the HEC-ResSim model.

Fig. A3. Taylor diagram showing the performance of rainfall downscaling during the validation period (1951-1975).

Fig. A4. Taylor diagram showing the performance of *T_min_* downscaling during the validation period (1951-1975).

Fig. A5. Taylor diagram showing the performance of *T_max_* downscaling during the validation period (1951-1975).

**List of Tables**

Table A1. Adopted list of GCMs for the present study

| Model | Country of origin | Institute | Spatial resolution |
| --- | --- | --- | --- |
| BCC-CSM1-1 | China | Beijing Climate Center | 2.8˚$\times$2.8 ˚ |
| GFDL-ESM2G | USA | Geophysical Fluid Dynamics Laboratory, USA | 2.5˚$\times$2.0 ˚ |
| MIROC5 | Japan | University of Tokyo | 1.4˚$\times$1.4 ˚ |
| MIROC-ESM-CHEM | Japan | University of Tokyo | 2.8˚$\times$2.8˚ |
| NorESM1-M | Norway | Meteorological Institute | 2.5˚$\times$1.875˚ |
| GFDL-CM3 | USA | Geophysical Fluid Dynamics Laboratory, USA | 2˚$\times$2 ˚ |
| IPSL-CM5A-LR | France | Institut Pierre Simon Laplace | 2˚$\times4$ ˚ |
| IPSL-CM5A-MR | France | Institut Pierre Simon Laplace | 1.25°$\times$2.5° |
| HadGEM2-ES | UK | Met Office, Hadley Centre | 1.2˚$\times$1.8 ˚ |
